# Therapy of spinal cord injury by zinc modified gold nanoclusters via immune-suppressing strategies

**DOI:** 10.1186/s12951-021-01035-8

**Published:** 2021-09-20

**Authors:** Sen Lin, Dan Li, Zipeng Zhou, Chang Xu, Xifan Mei, He Tian

**Affiliations:** 1 Department of Orthopedics, First Affiliated Hospital of Jinzhou Medical University, Jinzhou, People’s Republic of China; 2 Department of Basic Science, Jinzhou Medical University, Jinzhou, People’s Republic of China

**Keywords:** Spinal cord injury, Gold nanoclusters, Monocytes/macrophages, Polarization, Inflammation, Neurons, Apoptosis, Oxidative stress

## Abstract

**Background:**

Spinal cord injury (SCI) is damage to the central nervous system (CNS) that causes devastating complications from chronic pain to breathing problems. Unfortunately, few effective and safe treatments are known to relieve the damages of SCI. Nanomedicines are used for the treatment of SCI with relatively few side effects, but only depending on the delivery of additional drugs, which increase complexity to the treatment. Considering the urgent need for saving SCI patients, it is important to develop promising nanobiotechnology for relieving their pains.

**Methods:**

The clinical survey was used to investigate SCI patients, thereafter the therapy plan was designed. The receiver-operating characteristics (ROC) curves of the prediction model were built to find symptoms after SCI. The treatment plan (i.e. immunosuppressive strategy) was designed by manufacturing therapies based on gold nanoclusters (AuNCs). The response of the immune cells (macrophages) was studied accordingly. The western blot, reactive oxygen species (ROS) activity assay, enzyme-linked immunosorbent assay (ELISA), quantitative real-time PCR (RT-qPCR), and immunochemical staining were used for evaluation of the in vivo and in vitro therapeutic effects.

**Results:**

We found increased monocytes/macrophages (M/Ms) levels in 114 SCI subjects (44.7% with severe SCI complications) by the clinical survey. Additionally, the enhanced macrophage level was found to be closely related to the walking disorder after SCI. Since macrophages were central effector cells of the immune system, we assumed that the immune-suppressing strategies could be used for SCI therapy. Thereafter, AuNCs were stabilized by dihydrolipoic acid (DHLA) enantiomers (including DL-DHLA, R-DHLA; A racemic mixture (R and S) was denoted as DL; R and S refer to Rectus and Sinister), obtaining DL-DHLA-AuNCs and R-DHLA-AuNCs, respectively. In addition, zinc-modified DL-DHLA and R-DHLA stabilized AuNCs (i.e., DL-DHLA-AuNCs-Zn and R-DHLA-AuNCs-Zn) were investigated. Among these AuNCs, R-DHLA-AuNCs-Zn showed the most remarkable therapeutic effect for promoting the polarization of pro-inflammatory macrophages and reducing neuronal ROS-induced apoptosis and inflammation in vitro and in vivo; the lesion size was decreased and the survival rate of ventral neurons is higher.

**Conclusions:**

R-DHLA-AuNCs-Zn have comprehensive therapeutic capabilities, especially the immune-suppressing effects for the therapy of SCI, which is promising to relieve the pain or even recover SCI for the patients.

**Graphical abstract:**

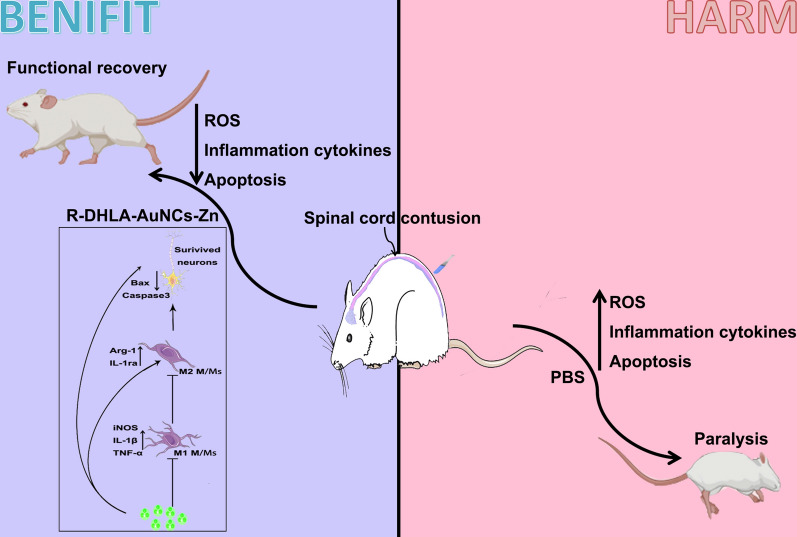

**Supplementary Information:**

The online version contains supplementary material available at 10.1186/s12951-021-01035-8.

## Background

A spinal cord injury (SCI), i.e., damage to the spinal cord or nerves at the end of the spinal canal, causes continuous neuroinflammation in the epicenter and surrounding areas of the injured focus, leading to chronic damage that affects an estimated 2.5 million people around the world [1]. Many treatments are investigated for relieving the symptoms of SCI [2–5]. For instance, steroid pulse therapy and neurotrophic rehabilitation relieve spinal cord ischemia, hypoxia and promote motor function. However, these strategies have side effects such as the high-frequency treatment. The efficiency for specific targeting treatment of the lesions is also insufficient. Anti-neuroinflammation drugs, such as glucocorticoids, minocycline, and ganglioside, have been used for the treatment of SCI [6]. The high dosages of these drugs are normally required, which cause side effects such as gastrointestinal haemorrhage, respiratory tract infection, and disability [7, 8]. Polarized macrophages are referred to as inflammatory (M1, i.e., classically activated) and regulatory (M2, i.e., alternatively activated) macrophages. It has been reported that monocytes/macrophages (M/Ms) gather in the lesion after SCI, the injured spinal cord predominantly exists M1 cytotoxic M/Ms [1, 3]. Exploring why the microenvironment induced by SCI plays a vital role in stimulating the polarization of M1, which is also important for the establishment of new neuroprotective therapies.

Nanomaterials have recently been used to treat SCI, based on drug delivery, reducing many side effects. However, the delivery process requires high costs and complicated protocols [4, 9–11]. The facile and low-harmful treatment strategies for SCI are still in great demand. Metal nanoclusters (NCs), having sizes (< 3 nm) smaller than traditional nanomaterials, are investigated as injected drugs for the therapy of some diseases owing to their low cytotoxicity, biocompatibility, high cleaning efficiency, and blood–brain barrier crossing ability [2]. As a representative of NCs, AuNCs are promising internal drugs due to their thermal stability, low toxicity, excellent biocompatibility in the physiological environment.

In this study, we used four types of NCs including DL-DHLA-AuNCs, R-DHLA-AuNCs, DL-DHLA-AuNCs-Zn, and R-DHLA-AuNCs-Zn to alleviate the damages after SCI. Several previous works used the reduced α- dihydrolipoic acid (i.e., α-DHLA or DL-DHLA) for stabilizing AuNCs [12, 13]. Compared to DL-DHLA, R-DHLA was more efficiently absorbed by cells [14]. We assumed R-DHLA stabilized AuNCs may have a desirable surface, enabling more efficient targeting effects. However, different from DL-DHLA, R-DHLA has not been particularly used to stabilize AuNCs or treat diseases. This was probably because their special surface tuning effect for AuNCs was ignored, though it might show an exciting effect for treating disease. Interestingly, we found that R-DHLA-AuNCs-Zn relieved activated M/Ms, reduced pro-inflammatory cytokines, and inhibited neuronal apoptosis without loading additional therapeutic agents. Compared with various organic drugs and nano-delivery systems [15], the functional recovery of SCI rats was most remarkable through the administration of R-DHLA-AuNCs-Zn. This indicated that chiral AuNCs modified with zinc were promising to treat SCI without being combined with additional drugs. Compared with previous nano-drug delivery systems, direct application of nano agents may be easier to popularize.

## Methods

### Chemical reagents and instrument

Anti-NeuN antibody, anti-CD11b antibody, anti-IL-1β antibody, anti-TNF-α antibody, anti-iNOS antibody, and anti-Arg-1 antibody were obtained from Abcam. Anti-Iba antibody was obtained from Wako. The Anti-Cleaved-Caspase-3 antibody and anti-Bax antibody were obtained from Cell Signaling Technology. The anti-β-Tubulin antibody and secondary antibodies were purchased from Proteintech. Transmission electron microscopy (TEM) and high-resolution transmission electron microscopy (HR-TEM) images were recorded using Tecnai™ G2 F30 Series. Energy-dispersive X-ray spectroscopy (EDS) was performed using JEM 2100F. Inductively coupled plasma mass spectrometry (ICP-MS) was used to determine the concentration of NCs. Zeta potential was studied by Mastersizer3000.

### Participants

SCI diagnosis was confirmed by spine surgeons from the first afflicted hospital of Jinzhou Medical University. People with communicable diseases or taking anti-inflammatory medications at the time were excluded. All 204 subjects were free of major medical issues and neurological conditions other than SCI. All investigated subjects provided informed consent to participate and the Review Board for the First Affiliation Hospital of Jinzhou Medical University approved the study. Eligibility for this study includes the American Spinal Injury Association (ASIA) Injury Scale classification with A, B, C, or D, the level of neurological damage between C4 and T12, and medical stability [16]. All SCI patients were in the post-acute stage 1–3 weeks after injury and receive standard treatment, including rehabilitation therapy tailored to their specific needs. All human study involving the analysis of medical record data was conducted according to the principles expressed in the Declaration of Helsinki and was approved by the Review Board of Jinzhou Medical University. Informed consent was obtained from each subject.

### Synthesis and characterization of NCs

For a typical synthesis of R-DHLA-AuNCs, 5.2 mg of R-lipoic acid was dissolved into 16 mL of water solution under aggressive super-sonications. A certain volume of 1 M NaOH was dropped until the complete dissolution. After that, 160 μL of HAuCl_4_ (50 mM) was injected with stirring. After 5 min, an aqueous solution of NaBH_4_ (200 μL, 0.1 M) was added slowly to the mixture and the stirring was continued for an additional 5 min. The mixture was kept overnight. DL-DHLA-AuNCs were synthesized the same way by using DL-lipoic acid as an alternative agent for R-lipoic acid. For the synthesis of zinc modified AuNCs, the same procedure was performed except 0.125 mM of ZnCl_2_ was added before the addition of NaBH_4_. The products were purified by a 3.5 kDa dialysis bag against pH 7.5 phosphate buffer saline (PBS) buffer solutions overnight.

### Cytotoxicity assay

RAW264.7 cells, Mouse Astrocytes-cerebellar (MA-c) cells, and Glial (Oligodendrocytic) Hybrid (MO3.13) cells were obtained with Dulbecco’s modified Eagle medium (DMEM) supplemented with 10% fetal bovine serum (FBS) plus 1% penicillin–streptomycin (PS). The cell toxicity of all NCs was determined using the 3-(4,5)-dimethylthiahiazo (-z-y1)-3,5-di-phenytetrazoliumromide (MTT) according to the manufacturer’s instructions. Cells were plated in 96-well plates at 1 × 10^4^ cells/well, respectively, and allowed to proliferate for 24 h, after which 20 μL of MTT solution was added to each well, followed by incubation for 2 h. The absorbance at 450 nm was measured using a microplate reader (Biotek, Winooski, VT, USA).

### Animal treatments

In this study, male Sprague–Dawley rats (weight 220–240 g) were used. Rats were fed in a controlled place with standard rodents. Animals were kept at 22 ± 1 °C, 12 h light, 12 h dark cycle. The study was permitted by the Jinzhou Medical University Review Board for the care of animals (NO. 2020009). A 2 mm diameter and 10 g impounder were filled on the T9–T10 spinal cord from 25 mm height, resulting in spinal cord moderate contusion. The bladder was massaged twice a day until bladder function regained normally. SCI group was intravenously injected with physiological saline, three times a day. R-DHLA-AuNCs-Zn group was intravenously injected with 100 μL of 50 μM R-DHLA-AuNCs-Zn after SCI, three times a day.

### Behavioral assessment

The behavioral assessment was measured by behavioral analysis using the Basso, Beattie, and Bresnahan (BBB) open-field locomotor test [17]. A double-blind assessment was used at 0, 1, 3, 7, 14, 21, and 28 days post-injury. BBB scores range from 0 to 21 points. 0 point revealed complete paralysis, and 21 points indicated normal function. The average scores were calculated by the grading standard in locomotion recovery after SCI. After surgery, the bladders were manually squeezed by applying pressure twice a day. We determined the relationship between pressure and urination ability, and grade bladder function from 0 to 3 (3 = dysfunction, high urination after the implementation of medium and high pressure; 2 = partial dysfunction, moderate urination after moderate pressure; 1 = mild dysfunction, squeeze out a small amount of urine after slight pressure; 0 = full function, no pressure required for urine discharge). This procedure continued until the bladder function of the rats recover.

### Staining and imaging of injured spinal cord in rats

For histological analysis, the rats were sacrificed and tissues were collected and fixed in 4% buffered formaldehyde. Spinal cord sections were then paraffin-embedded, sectioned, stained with hematoxylin and eosin (H&E), and analyzed with ImageJ. Spinal cord slices were incubated with primary antibody at 4 °C overnight, washed with 1 × PBS 3 times, and then incubated with secondary antibody (1:500, Alexa Fluor 488 or Alexa Fluor 568, Invitrogen) for 2 h at room temperature. Cell nuclei were incubated with DAPI solution (1:1000) dyeing. All slides were investigated under a fluorescence microscope.

### RT-qPCR

The injured spinal cord tissue was collected from the time point for the experiment of RT-qPCR. The relative expression levels of the target genes were normalized to those of the housekeeping gene ribosomal protein S18 (RPS18) and the target genes from the experimental group were compared with the corresponding target genes from the control group using the (1 + e)^−ΔΔCT^ method [18]. The relative oligonucleotide primers were listed in Additional file 1: Table S1.

### Western blot

At 7 days post-operation, after anesthetized, the spinal cord (1 cm from the center of the injury point) was taken. Tissues were chopped into small chunks with fine scissors and then dissolved in RIPA lysis buffer (Beyotime, China). The final protein concentration was quantified by BCA Protein Kit (Beyotime, China), using bovine serum albumin (BSA) as the protein standard. The same amount of protein samples was added to polyacrylamide gels. The samples were added to SDS-PAGE and transferred to a membrane, then blocked with 1% BSA in TBST at room temperature for 2 h. The membranes were immersed with the primary antibodies at 4 °C overnight. On the second day, the membranes were incubated with the secondary antibodies at room temperature for 2 h. The membranes were developed using ItTMTS2 Imager (UVP, LLC, Upland, CA, USA), and relative optical density was analyzed by ImageJ2x software (National Institute of Health, Bethesda, MD, USA).

### ROS Scavenging and ELISA assays

The spinal cord tissue was weighed, washed with pre-cooled PBS, and homogenized. Quantitative analysis of TNF-α and IL-1β levels in the ischemic penumbra was conducted with ELISA assays [19].

### Enzyme-like activity of R-DHLA-AuNCs-Zn

The CAT-like, GPx-like, and SOD-like activities of R-DHLA-AuNCs-Zn were determined by assay kits (Beyotime Bioengineering Institute) according to the introduction [20]. In the CAT-like assay protocol, the CAT-like sample reacts with hydrogen peroxide (H_2_O_2_) to produce water and oxygen. The residual H_2_O_2_ reacted with a probe to produce a colored product with an OD of 570 nm. Therefore, the CAT-like activity of R-DHLA-AuNCs-Zn is reversely proportional to the signal obtained.

In the GPx-like assay protocol, GPx oxidizes glutathione (GSH) to produce glutathione disulfide (GSSG). Glutathione reductase (GR) then reduces the GSSG to produce GSH, which also consumes nicotinamide adenine dinucleotide phosphate (NADPH). The decrease of NADPH is proportional to GPx activity, which can be monitored at OD = 340 nm.

The SOD-like activity was measured based on the xanthine-xanthine oxidase, which was used to generate O2·− . The nitroblue tetrazolium (NBT) reduction was used as an indicator of O2·− production. SOD competed with NBT for O2·− ; The percentage of inhibition of NBT reduction showed the amount of SOD present.

### Statistical analysis

The data were expressed as mean ± standard deviation and analyzed by SPSS 19.0. Student's t-test and one-way ANOVA were used to compare the data between two and more groups. In addition, Mann–Whitney U was used to test the BBB. Wilcoxon rank-sum test was used to compare the median of data from RT-qPCR analysis, histogram analysis, and fluorescence. The difference was considered to be statistically significant, with a value of P < 0.05.

## Results

### The clinical hematology panel of SCI subjects

**Fig. 1:**
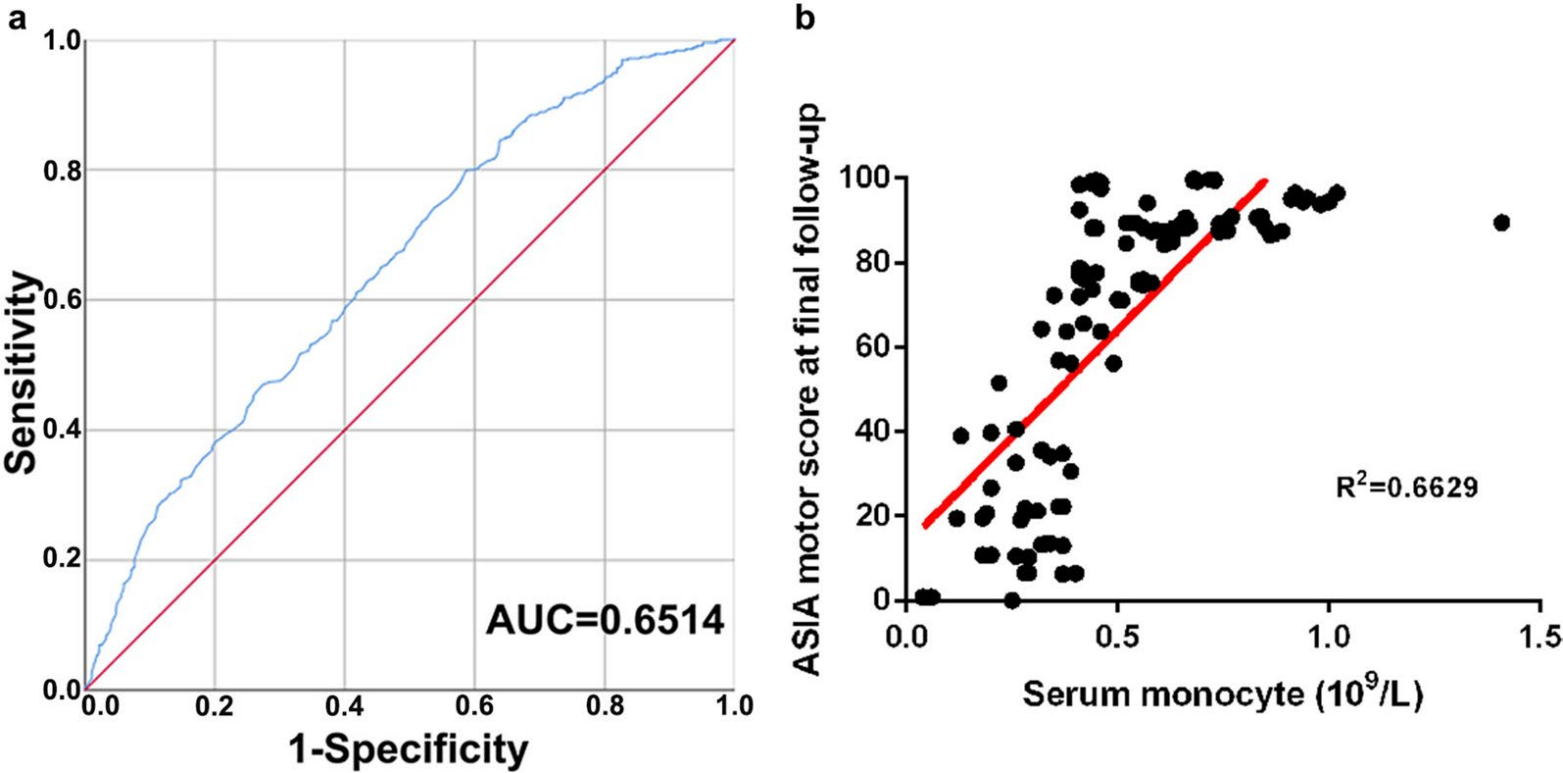
M/Ms levels predict the functional prognosis after SCI in humans. **A** The receiver-operating characteristics (ROC) curves of the prediction model based on admission M/Ms concentrations for discriminating the walkability. **B** Scatter plots illustrating the correlations between the American Spinal Injury Association (ASIA) motor score at final follow-up and the serum monocytes/macrophages concentration at admission

Participants were collected from 114 SCI subjects and 90 healthy people as control from the First Affiliation Hospital of Jinzhou Medical University from October 2018 to December 2020. The data about controls' and patients' demographic and clinical characteristics was summarized. According to Table 1, the levels of lymphocytes, eosinophils, and basophils in subjects with paraplegia or quadriplegia were not significantly different from those in the healthy group (control group), while red blood cells (RBCs), white blood cells (WBCs), neutrophils and monocytes were significantly increased in SCI subjects with paraplegia or quadriplegia. Acute SCI patients had significantly enhanced white blood cell levels (P < 0.05), neutrophils levels (P < 0.05), and monocyte levels (P < 0.05) compared to the control group (Table 1). And it had no significant difference in subjects with sex, age, and ASIA in patients compared to the control group (Additional file 1: Table S2). This result suggested that blood-derived leukocytosis was one of the hematology panel manifestations of SCI [1, 3]. Next, the monocyte levels were investigated during the acute phase of SCI. It was found that the M/Ms level had a close relationship with the walking ability (area under the curve (AUC) = 0.6514, 95% confidence interval 0.807–0.996, p < 0.0001) (Fig. 1a) after SCI. The relationships between the M/Ms level and the ASIA motor score at final follow-up were studied and there was a positive correlation between the M/Ms levels at admission and the ASIA motor score at final follow-up (R^2^ = 0.6629) (Fig. 1b). These results showed that the acute M/Ms level was closely related to the functional prognosis after human SCI. On the other hand, the healthy subjects had relatively low M/Ms levels. Based on these results, it was worth wondering whether the suppressing of the M/Ms level could relieve the symptoms of SCI. Thereafter, several NCs based agents were developed to regulate the M/Ms levels.

**Table 1:** Blood chemistry of subjects

Subjects	N/(n)	RBCs (10^12^/L)	WBCs (10^9^/L)	Neutrophils (10^9^/L)	Lymphocytes (10^9^/L)	Monocytes (10^9^/L)	Eosinophils (10^9^/L)	Basophils (10^9^/L)
SCI patients	114	4.0 ± 0.7*	10.1 ± 4.6*	8.0 ± 4.8*	1.4 ± 0.8	0.52 ± 0.24*	0.06 ± 0.09	0.01 ± 0.02
Paraplegia	63	4.0 ± 0.6*	9.6 ± 3.2*	7.5 ± 3.3*	1.4 ± 0.8	0.50 ± .23*	0.06 ± 0.09	0.01 ± 0.02
Tetraplegia	51	3.9 ± 0.8*	10.4 ± 5.4*	8.4 ± 5.6*	1.5 ± 0.8	0.52 ± 0.26*	0.06 ± 0.10	0.01 ± 0.02
Control	90	4.5 ± 0.5	6.1 ± 2.8	3.9 ± 2.0	1.6 ± 0.6	0.45 ± 0.23	0.06 ± 0.10	0.01 ± 0.05

RBCs, red blood cells; WBCs, white blood cells. *P < 0.05

### Characterization of the NCs

**Fig. 2:**
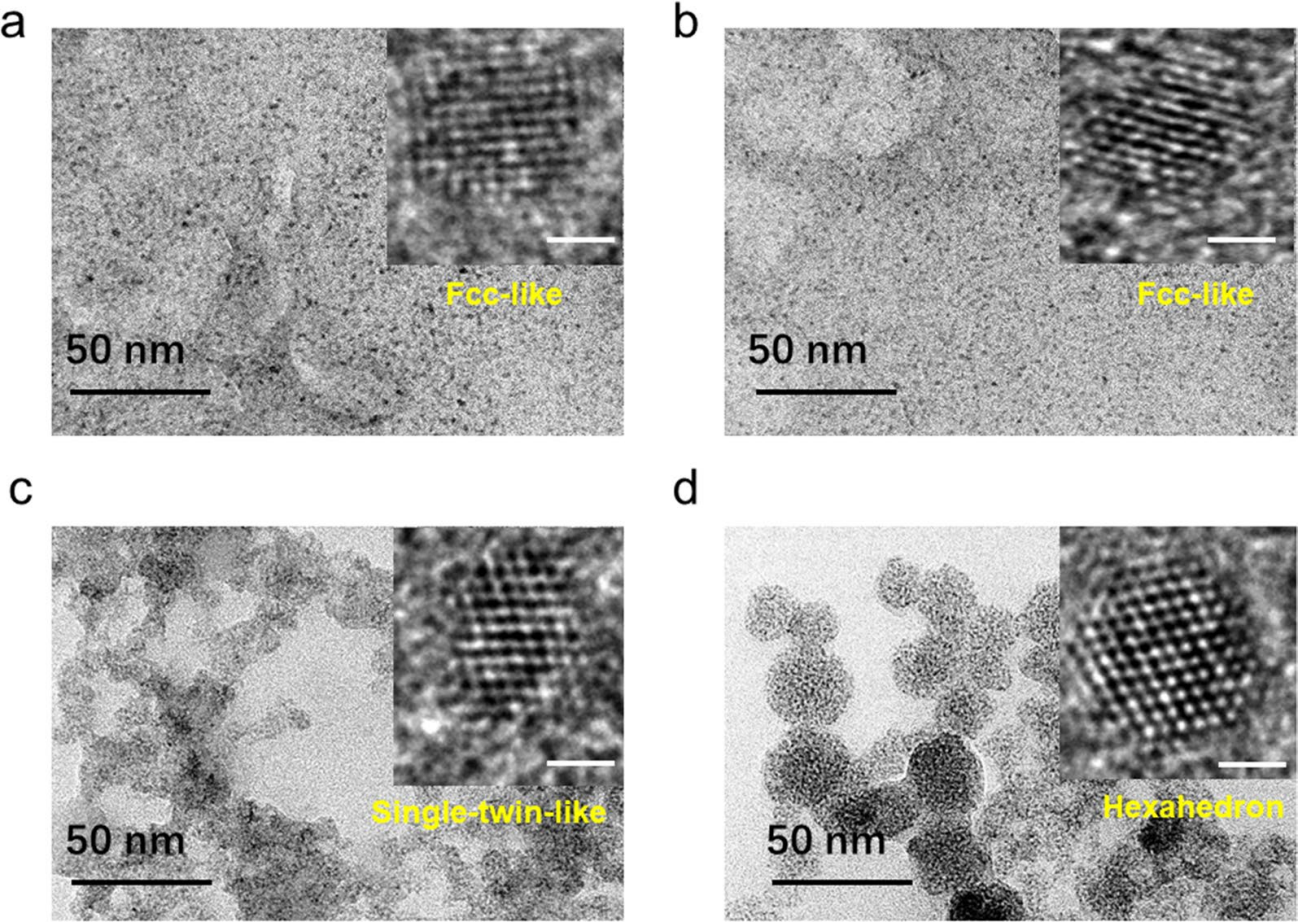
TEM of DL-DHLA-AuNCs (**a**), R-DHLA-AuNCs (**b**), DL-DHLA-AuNCs-Zn (**c**), R-DHLA-AuNCs-Zn (**d**); The inset show the HR-TEM of these samples and the scale bar is 1 nm

R-DHLA-AuNCs and DL-DHLA-AuNCs with and without the modification of zinc were characterized by TEM and HR-TEM (Inset) (Fig. 2). The results showed that DL-DHLA-AuNCs and R-DHLA − AuNCs have small sizes (< 3 nm), which are consistent with our previously reported AuNCs [2, 21]. DL-DHLA-AuNCs-Zn were irregularly aggregated and the entire size was large. R-DHLA-AuNCs-Zn were assembled into relatively regular and well-dispersed spheres. Zinc-modified AuNCs can produce larger particles, or redisperse into small clusters [19]. Therefore, zinc-modified AuNCs can have the advantages of large particles and can also produce small size effects [22]. TEM-EDS (Additional file 1: Fig. S1 and Table S3) showed that the atomic ratio of zinc in R-DHLA-AuNCs-Zn was greater than that in DL-DHLA-AuNCs-Zn, indicating that compared with DL-DHLA-AuNCs-Zn, zinc played a more important role in the products of R-DHLA-AuNCs-Zn. In the inset of Fig. 2, the HR-TEM shows the lattice of the clusters. We can find fcc-like particles of about 2.4 nm in Fig. 2a, b for DL-DHLA-AuNCs and R-DHLA-AuNCs, with a characteristic fast Fourier transform following the typical AuNCs [20]. The structure of DL-DHLA-AuNCs-Zn identified in the inset of Fig. 2c can be associated with a single twin particle of about 2.4 nm. The size was not significantly larger than AuNCs. On the other hand, much larger clusters produced were identified in the R-DHLA-AuNCs-Zn sample (Fig. 2d, inset). We can observe a hexahedron particle of about 2.8 nm, which is associated with the zinc-modified AuNCs. The larger size of R-HDLA-AuNCs-Zn observed from HR-TEM images also clearly showed that more zinc was modified on R-DHLA-AuNCs.

Compared to the AuNCs, the zinc-modified AuNCs had two prominent zeta potential peaks (Additional file 1: Figure S2a, b). This indicated the samples may have smaller and larger size species, corresponding to the assembled AuNCs and dispersed AuNCs respectively. Electrospray ionization mass spectrometry (ESI–MS) was used for further understanding the surface condition (Additional file 1: Figure S2c, d) of the NCs. There was no significant difference between the ESI–MS of DL-DHLA-AuNCs and DL-DHLA-AuNCs-Zn. At the same time, compared with R-DHLA-AuNCs, we observed more zinc in R-DHLA-AuNCs-Zn samples, indicating that relatively more zinc was modified on R-DHLA-AuNCs. This is consistent with the TEM-EDS and HR-TEM studies.

### Toxicity and anti-inflammatory effect in vitro

**Fig. 3:**
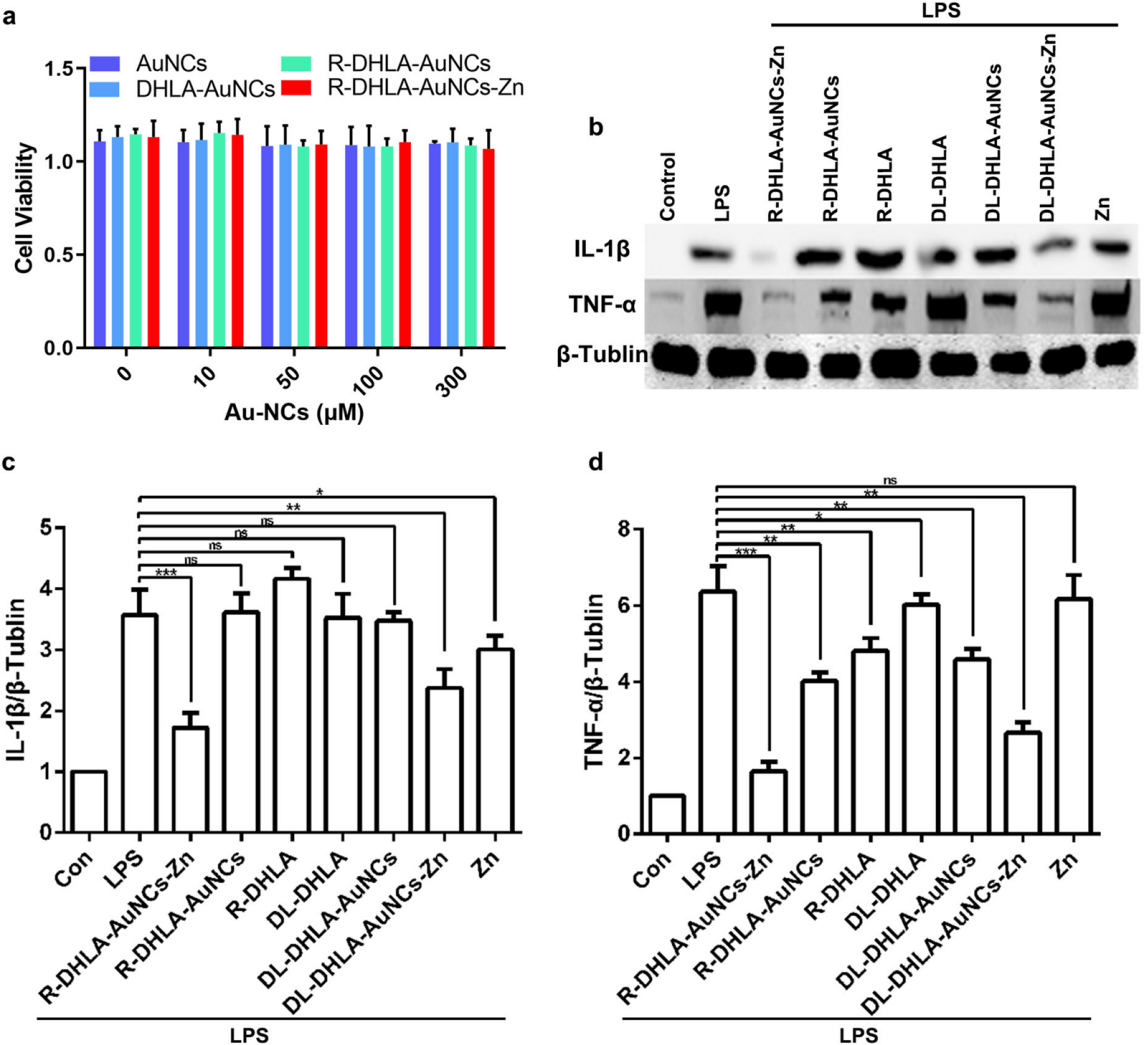
Comparison of the cytotoxic effect and anti-inflammatory effect of NCs to RAW264.7, including DL-DHLA-AuNCs, R-DHLA-AuNCs, DL-DHLA-AuNCs-Zn, and R-DHLA-AuNCs-Zn: **a** RAW264.7 cells. **b** The western blot analysis (**b**) and semiquantitative analysis of IL-1β (**c**) and TNF-α (**d**) *P < 0.05;**P < 0.01;***P < 0.001; Data presented as mean ± SD. (n = 6 /group); Con indicated the culture of only RAW264.7 cells after 24 h; LPS represented the RAW264.7 cells were tested by LPS-stimulation after 24 h; Others represented RAW264.7 cells were pre-stimulated by LPS and then cultured with various agents after 24 h

The viability of RAW 264.7 cells was investigated in the absence and presence of NCs based on the MTT experiments (Fig. 3a). After RAW 264.7 cells were treated 24 h, the cell viability was near 100% with various NCs, including DL-DHLA-AuNCs, R-DHLA-AuNCs, DL-DHLA-AuNCs-Zn, and R-DHLA-AuNCs-Zn, at various concentrations from 0–300 μM. The MTT assay demonstrated that all these NCs showed good biocompatibility and insignificant toxicity to macrophages even at high dosage in vitro, which was in good agreement with the previously reported NCs as safe injected drugs [23]. The patients after SCI have elevated levels of proinflammatory cytokines including interleukin 1 beta (IL-1β) and tumor necrosis factor-alpha (TNF-α), which indicated the inflammatory response [24]. Then, the inflammatory response was studied in lipopolysaccharide (LPS)-stimulated RAW 264.7 macrophage cells, and anti-inflammatory effects of NCs (50 μM) were studied after 24 h by western blot. As shown in Fig. 3b and c, IL-1β secretion was significantly enhanced after LPS stimulation of RAW 264.7 cells as compared to the control group. Zinc did not significantly influence TNF-α after LPS stimulation (Fig. 3b and d). The inhibition efficiency was only about 20%. R-DHLA, DL-DHLA, R-DHLA-AuNCs, and DL-DHLA-AuNCs slightly down-regulated the expression of TNF-α from about 30% to 70%. It was worth noting that R-DHLA-AuNCs-Zn significantly inhibited IL-1β and TNF-α after LPS stimulation with an efficiency of 85%. This indicated that R-DHLA-AuNCs-Zn had a stronger anti-inflammatory effect than other systems. Based on the above analysis, we believed that R-DHLA-AuNCs-Zn may have immunosuppressive effects and can be used for further treatment of SCI.

### Antioxidant effect of R-DHLA-AuNCs-Zn in vitro

**Fig. 4:**
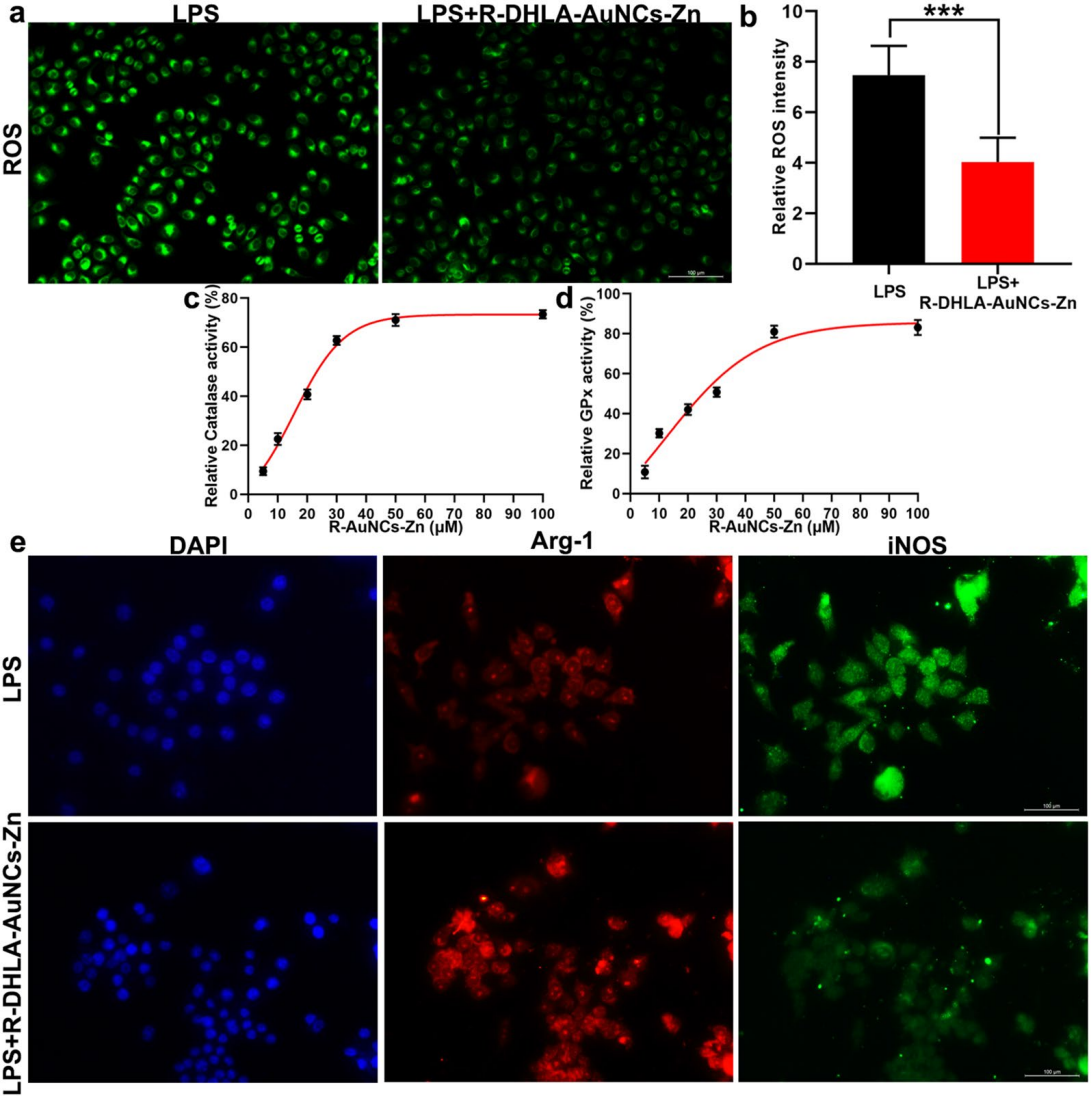
The anti-inflammatory effect of R-DHLA-AuNCs-Zn via altering local M1/M2 subsets. ROS staining (**a**) and representative quantification of ROS (**b**) in RAW264.7 cells treated with LPS or LPS + R-DHLA-AuNCs-Zn. The CAT-like (**c**) and GPx-like (**d**) activities of R-DHLA-AuNCs-Zn after stimulation of RAW264.7 by LPS. The immunofluorescence staining (**e**) of iNOS and Arg-1 in RAW264.7 cells treated with LPS or LPS + R-DHLA-AuNCs-Zn. ***P < 0.001. Scale bar = 100 μm. Data presented as mean ± SD. (n = 6 /group)

Oxidative stress or ROS is a hallmark of injury of SCI. Alleviating ROS level is considered an effective way of therapeutic intervention of SCI [25]. After 24 h of co-culture with R-DHLA-AuNCs-Zn, the ROS staining (Fig. 4a, b) of RAW 264.7 showed that R-DHLA-AuNCs-Zn remarkably inhibited ROS production. It was reported that AuNCs based materials might show enzyme-like properties after modifications, which suppressed the oxidant stresses [26]. To deeply understand the antioxidant reason, the CAT-like and GPx-like activities of R-DHLA-AuNCs-Zn were studied. The results showed that R-DHLA-AuNC-Zn exhibited multi-enzyme activities including both the CAT-like and GPx-like activities and the activities increased as a function of the concentrations, reaching a maximum value at 50 μM (Fig. 4c, d). Therefore, 50 μM of R-DHLA-AuNCs-Zn that showed excellent anti-oxidant effects were used for further treatment.

Expression of iNOS and Arg-1 is considered as M1 and M2 markers of immune cell polarization [26]. Therefore, the iNOS and Arg-1 were investigated by immunofluorescence. The ratio of iNOS^+^ cells (M1) was significantly decreased by treating with R-DHLA-AuNCs-Zn (Fig. 4e). Meanwhile, the ratio of Arg1^+^ cells (M2) was significantly increased (Fig. 4e). On the other hand, the expression of iNOS was significantly reduced after R-DHLA-AuNCs-Zn treatment, accompanied by the up-regulation of Arg-1 expression, indicating that R-DHLA-AuNCs-Zn changed the local M1/M2 subgroups and increased beneficial M/Ms subset (M2 type) ratio after overactivation. On the other hand, the R-DHLA-AuNCs-Zn group insignificantly influenced the cell viability of other glial cells and the inflammatory conditions (Additional file 1: Fig. S3). Therefore, M/Ms may mediate the inflammatory by the transformation of the polarization state [27, 28].

The polarization of M/Ms after LPS stimulation and LPS + R-DHLA-AuNCs-Zn treatment (Additional file 1: Fig. S4a) were investigated. The RAW 264.7 cells were stained with paired FITC-labeled iNOS and PI-labeled Arg-1. The percentage of M1 cells increased to about 61.89% (FITC-positive and PI-negative) after LPS stimulation, and ≈0.05% RAW 264.7 cells were at the M2 stage (PI-positive and FITC-negative; Fig. 4i). When RAW 264.7 cells were incubated with R-DHLA-AuNCs-Zn, the number of PI-positive and FITC-negative cells increased significantly (Additional file 1: Fig. S4b). These results indicate that R-DHLA-AuNCs-Zn protects M/Ms from LPS-induced inflammation. This result further suggested that the polarization from M1 toward M2 state after treating by R-DHLA-AuNCs-Zn had a relationship with the recovery of SCI.

### Neuroprotective effect of R-DHLA-AuNCs-Zn in vivo

**Fig. 5:**
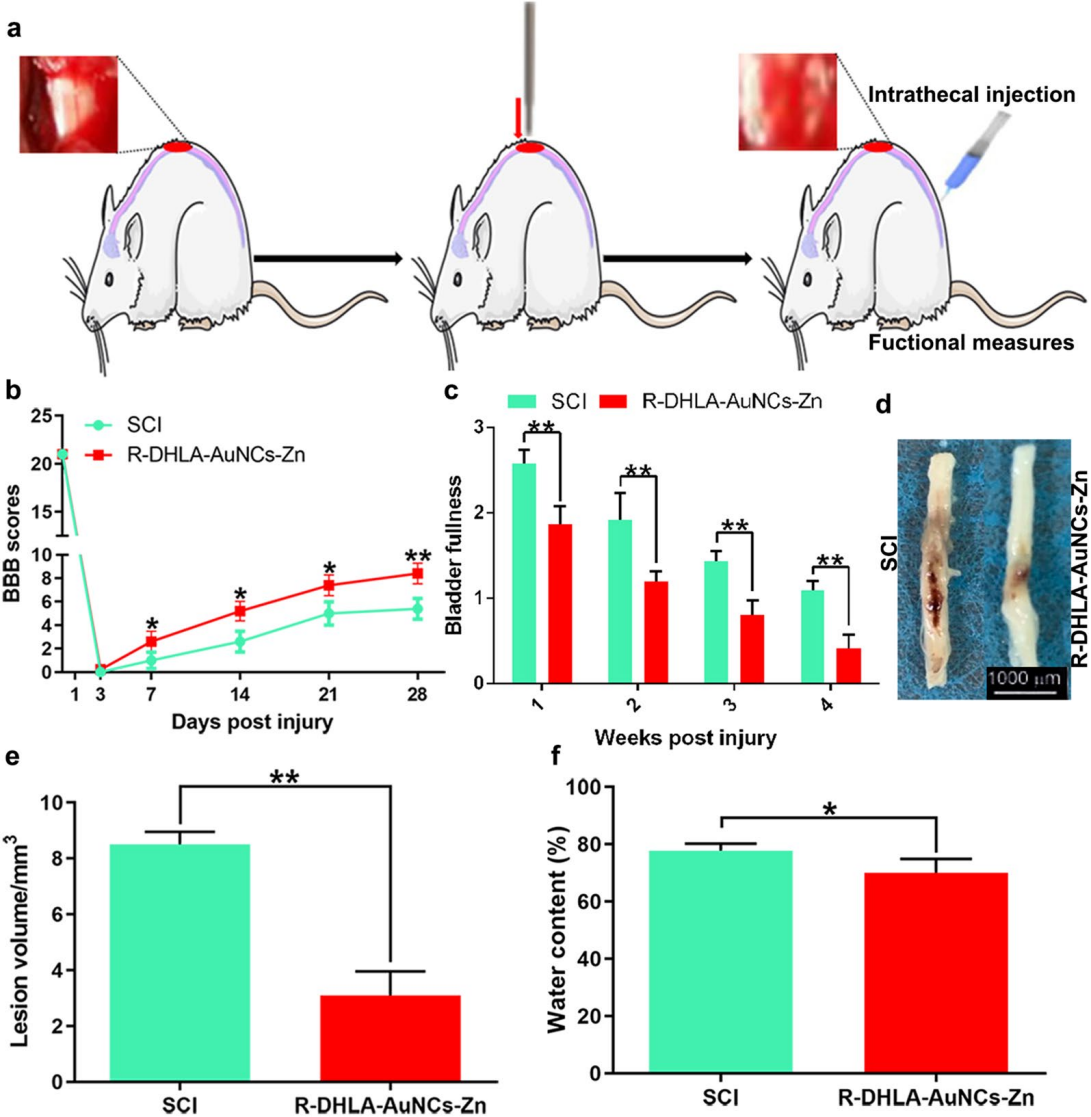
Morphological evaluation of the injured spinal cord and locomotor functional assessment. The experimental model for rats (**a**). Representative BBB scores of rats from R-DHLA-AuNCs-Zn group at different time points (**b**) (n = 10, *p < 0.05, **p < 0.01 compared to SCI group). Representative bladder assays (**c**) of rats from R-DHLA-AuNCs-Zn group at different time points (n = 10, *p < 0.05, **p < 0.01 compared to SCI group). Images of the spinal cord of SCI rat model and R-DHLA-AuNCs-Zn adminstration groups (**d**). Quantification of the lesion area percentage (**e**) in two groups (n = 6,**p < 0.01 compared to SCI group). Quantification of the water content (**f**) in two groups (n = 6,**p < 0.01 compared to SCI group). **P < 0.01;***P < 0.001. Data presented as mean ± SD. (n = 6 /group)

The neuroprotective effect of R-DHLA-AuNCs-Zn administration in SCI was studied using the rats model (Fig. 5a). We used a 28-day behavioral assessment and histopathological assessment to determine the treatment of SCI through BBB score and bladder filling conditions. After 3 days, BBB scores of the SCI group and the R-DHLA-AuNCs-Zn group showed no significant difference (Fig. 5b). Compared with the SCI group, the R-DHLA-AuNCs-Zn group showed a much higher BBB score and significantly reduced bladder filling measurement from 7 dpi (Fig. 5c), indicating R-DHLA-AuNCs-Zn significantly improved the recovery of hindlimb motor function during the acute course of SCI. As shown by the gross morphology of the injured spinal cords, the traumatic lesion area (red-colored region) on the spinal cord was visible (Fig. 5d). After treatment with R-DHLA-AuNCs-Zn, the lesion area was notably smaller than that of the SCI group without treatment. Nissl staining was used to identify traumatic lesion cavities by cell damage. Four weeks after injury, the gain of healthy tissue on the injured spinal cord was observed (Fig. 5e), reflecting R-DHLA-AuNCs-Zn significantly decreased the lesion volume. The water content was significantly lower in the R-DHLA-AuNCs-Zn administration group after SCI than in the control group (Fig. 5f). Compared with the various previously reported drugs [15], R-DHLA-AuNCs-Zn had a better therapeutic effect on SCI rats because the damage recovered faster (Table 2).

**Table 2:** Comparison of the motor function recovery after SCI by using different medicines

Nanomedicines	Motor function recovery	Therapeutic effects	Advantages/*disadvantages*	Refs.
Glutathione	Improve significantly	Antioxidant	Reduces inflammation; *very low bioavailability*	[29]
Methylprednisolone	Improve significantly	Immunosuppressant	Reduces inflammation; *water-sodium retention, susceptible to infection*	[6, 30]
Hydralazine	Improve significantly	Antioxidant	Reduces inflammation; *short half-life and long-term toxicity*	[31]
NT3-Chitosan	Improve significantly	Anti-infection, neuroprotection	Regeneration; *difficult to be dissolved in human fluid*	[32]
CeO_2_-PCL	Improve in vitro biocompatibility and auto-recovery abilities	Delivered a bone regeneration drug	Increases biocompatibility of the drug; *not available for injection and the in vivo effects are unknown*	[33]
Se-CQDs	Improve significantly after 8 weeks	Reduced the inflammation, astrogliosis, and apoptosis induced by secondary injury	Remarkable protective effect for nerves; *not cost-effective, toxic concern*	[34]
R-DHLA-AuNCs-Zn	Improve significantly after 7 days	Immunosuppressant	Reduces inflammation, anti-apoptosis, anti-oxidant, injectable, low toxicity, cost-effective, simple preparation	Current work

NT3, neurotrophin3; CeO_2_-PCL (CeO_2_ particles assembled onto poly (∊ -caprolactone) (PCL)) Se-CODS (Selenium-Doped Carbon Quantum Dots)

### Anti-inflammatory effect of R-DHLA-AuNCs-Zn in vivo

**Fig. 6:**
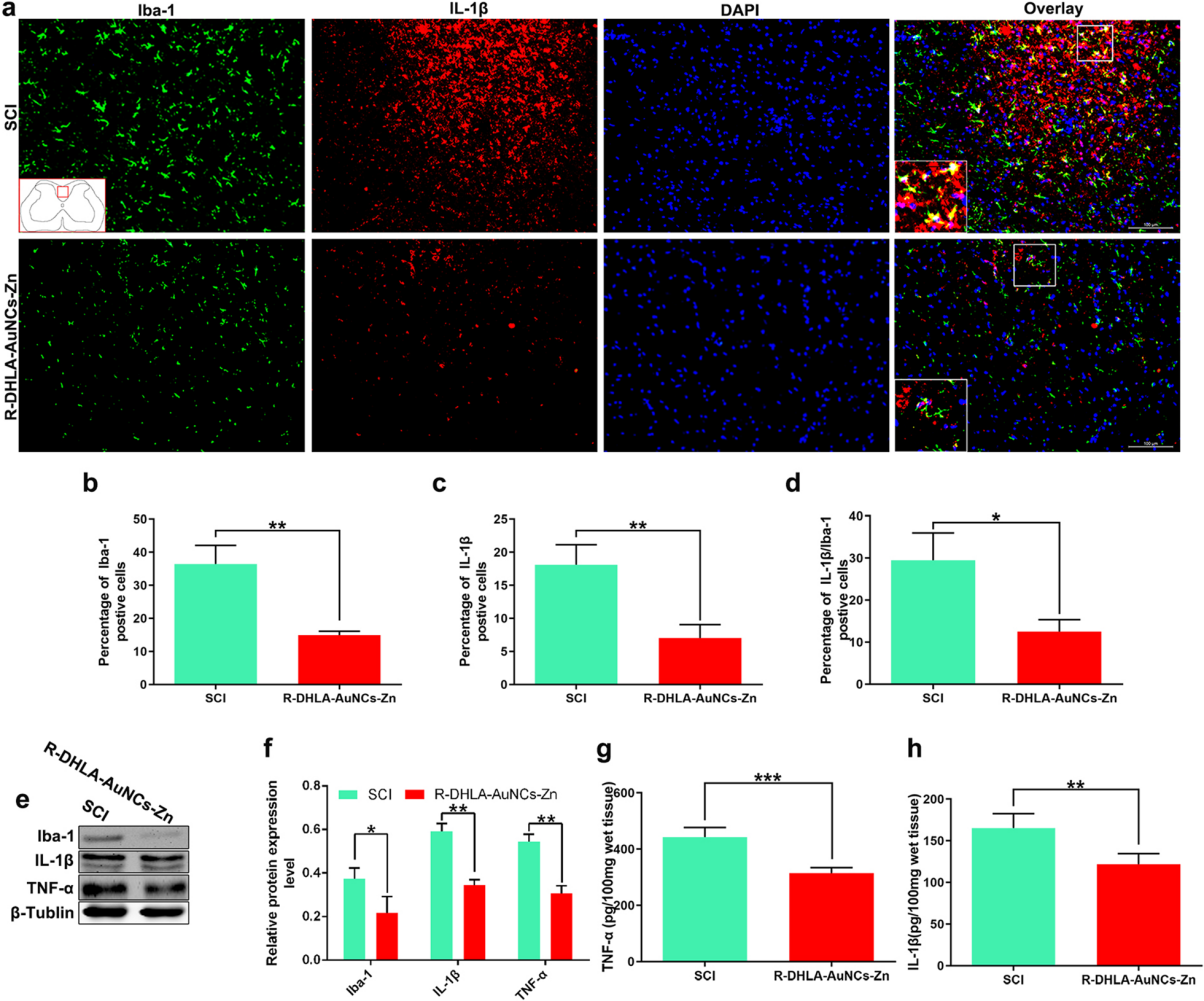
R-DHLA-AuNCs-Zn downregulated production of proinflammatory cytokines after SCI. The percentage of Iba-1 (**b**), IL-1β (**c**), and double-positive Iba-1/IL-1β (i.e., both Iba-1 and IL-1β were positive) (**d**) were investigated by immunofluorescence analysis (**a**). The levels of Iba-1, IL-1β, and TNF-α were shown in the western blot (**e**) and the corresponding semiquantitative analysis (**f**). The levels of TNF-α (**g**) and IL-1β (**h**) were studied by ELISA assays. Scale bar = 100 μm. *P < 0.05;**P < 0.01. Data presented as mean ± SD. (n = 6/group)

Inflammatory cytokines released from injured nerve cells and the spinal cord edema after trauma was reported in SCI patients [4]. Damaged tissues lead to the enhancement of inflammatory cytokines (e.g. IL-1β) and apoptotic proteins (e.g. Cleaved-caspase-3) [3, 35, 36]. Then, these factors were investigated by RT-qPCR. IL-1β and TNF-α were found to be significantly decreased in the R-DHLA-AuNCs-Zn treatment group (Additional file 1: Fig. S5a). On the other hand, the levels of Arg-1 and IL-1ra, which represented the protected effect for motor neurons [37], were highly increased after treating with R-DHLA-AuNCs-Zn, compared to the SCI group (Additional file 1: Fig. S5b). Expression and distribution of cytokines in the spinal cord were studied by immunofluorescence, western blot, and ELISA assays (Fig. 6). Compared to the SCI rats injected with only PBS after 7 days (Fig. 6a (a1, a2, a4)), significant reductions in the number of positive Iba (Fig. 6a (a5), b), positive IL-1β (Fig. 6a (a6), c), and double-positive Iba/IL-1β (Fig. 6a (a8), d) in the traumatic lesion area were observed with R-DHLA-AuNCs-Zn treatment using immunofluorescence analysis. R-DHLA-AuNCs-Zn administration also significantly suppressed M/Ms in the injured spinal cord as compared to SCI rats (Additional file 1: Fig. S6). The western blot analysis results were highly consistent with the immunohistochemical studies (Fig. 6e, f). In addition, the administration of R-DHLA-AuNCs-Zn reduced the levels of Iba-1 (Fig. 6g) and TNF-α (Fig. 6h), which were found by the ELISAs. All these results indicated that R-DHLA-AuNCs-Zn reduced the inflammatory response in SCI lesions.

### Anti-apoptotic effect of R-DHLA-AuNCs-Zn in vivo

**Fig. 7:**
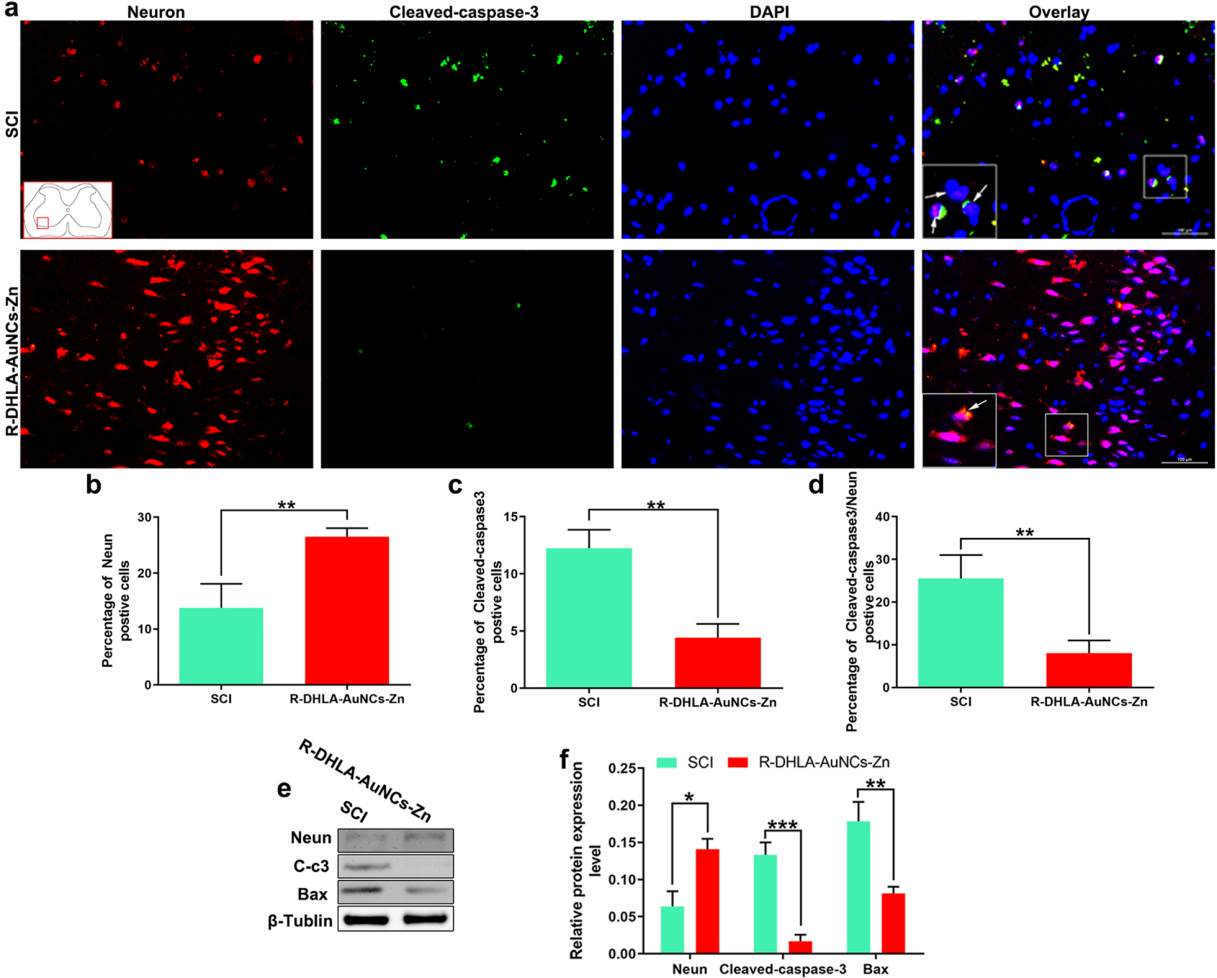
R-DHLA-AuNCs-Zn inhibited the neuron apoptosis after SCI by immunofluorescence analysis (**a**) and the percentage of NeuN (**b**), cleaved-caspase-3 (**c**), and cleaved-caspase-3/NeuN (**d**); Scale bar = 100 μm. The levels of NeuN, cleaved-caspase-3, and Bax by western blot analysis and semiquantitative analysis (**f**). *P < 0.05;**P < 0.01. Data presented as mean ± SD. (n = 6/group)

Previous studies suggested that ROS could induce neuron apoptosis and neuroinflammation. Overactivated apoptosis could inhibit nerve function recovery after CNS disorders [27, 38]. Since the content of cleaved caspase-3 is directly proportional to the degree of apoptosis, the cleaved caspase-3 in the spinal cord was detected by immunofluorescence and western blot (Fig. 7). The number of cleaved caspase-3^+^ and Neun^+^ cells counted on the ventral horn was significantly reduced in the R-DHLA-AuNCs-Zn administration group as compared to the SCI group (Fig. 7a–d). Moreover, the level of Bax in the ventral spinal cord was significantly reduced in the R-DHLA-AuNCs-Zn group as compared with that in the SCI group (Fig. 7e). Compared to the SCI group, R-DHLA-AuNCs-Zn administration induced an increase in neuron expression (Fig. 7f). All these results indicated that R-DHLA-AuNCs have an anti-apoptotic effect, which plays an important role in the therapy of SCI.

Abnormally elevated ROS may cause neuroinflammation and apoptosis [27, 38]. Therefore, the ROS change after the administration of R-DHLA-AuNCs-Zn was investigated via ROS staining. We found that R-DHLA-AuNCs-Zn administration decreased ROS level on the 7th day after SCI (Additional file 1: Fig. S7a–b). Meanwhile, the ROS-induced lipid peroxidation [37], including 4-hydroxynonenal (HNE) (Additional file 1: Fig. S7c) and malondialdehyde (MDA) (Additional file 1: Fig. S7d) were reduced. Simultaneously, GSH was (Additional file 1: Fig. S7e) was found to be promoted. R-DHLA-AuNCs may prevent lipid peroxidation by reducing oxidized glutathione, thus enhanced the GSH level [38]. On the other hand, the SOD-like activity [39], which is also important for antioxidation, significantly rose (Additional file 1: Fig. S7f). These results indicated the antioxidant effects of R-DHLA-AuNCs-Zn, which were in good consistent with the result in vitro (Fig. 4).

### The recovery of SCI by the administration of R-DHLA-AuNCs-Zn in vivo

**Fig. 8:**
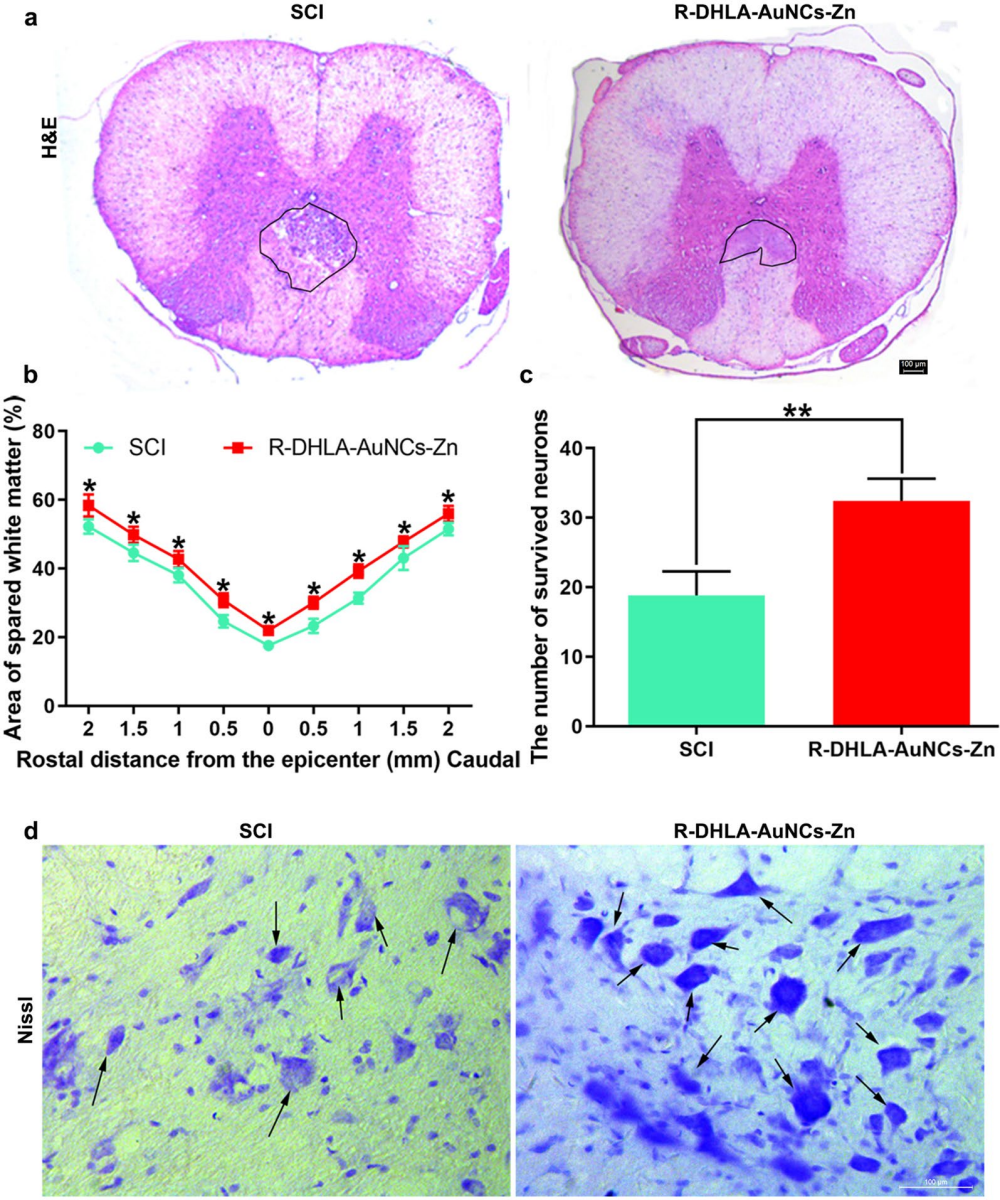
R-DHLA-AuNCs-Zn exerted a nerve recovery effect after SCI. The lesion of the spinal cord was studied by H&E staining (**a**) and semiquantitative analysis (**b**). The semiquantitative analysis (**c**) and the images (**d**) of surviving neurons in Nissl staining. Scale bar = 100 μm. *P < 0.05;**P < 0.01. Data presented as mean ± SD. (n = 6/group)

We investigated the histological changes of the SCI rats after the administration of R-DHLA-AuNCs-Zn. The cavity area around the epicenter at 4 weeks after SCI via H&E staining was exhibited in Fig. 8a. Specifically, the cavity area increased at the 28th-day post-injury after R-DHLA-AuNCs-Zn administration, adjacent to the lesion area for the SCI group. This result showed that R-DHLA-AuNCs-Zn promoted the white matter area from the head to the tail after SCI (Fig. 8b). In addition, compared with the SCI group, the number of ventral horn neurons in the R-DHLA-AuNCs-Zn group was significantly increased (Fig. 8c). By Nissl staining, we found that R-DHLA-AuNCs-Zn promoted the survival of neurons in the ventral horn 4 weeks after SCI (Fig. 8d). In addition, the injured spinal cord showed a decrease in total tissue volume around the epicenter. Therefore, R-DHLA-AuNCs-Zn treatment continued to inhibit secondary reactions and reduced the severity of the initial reactive inflammatory response after SCI.

### Immune response after the administration of R-DHLA-AuNCs-Zn in vivo

**Fig. 9:**
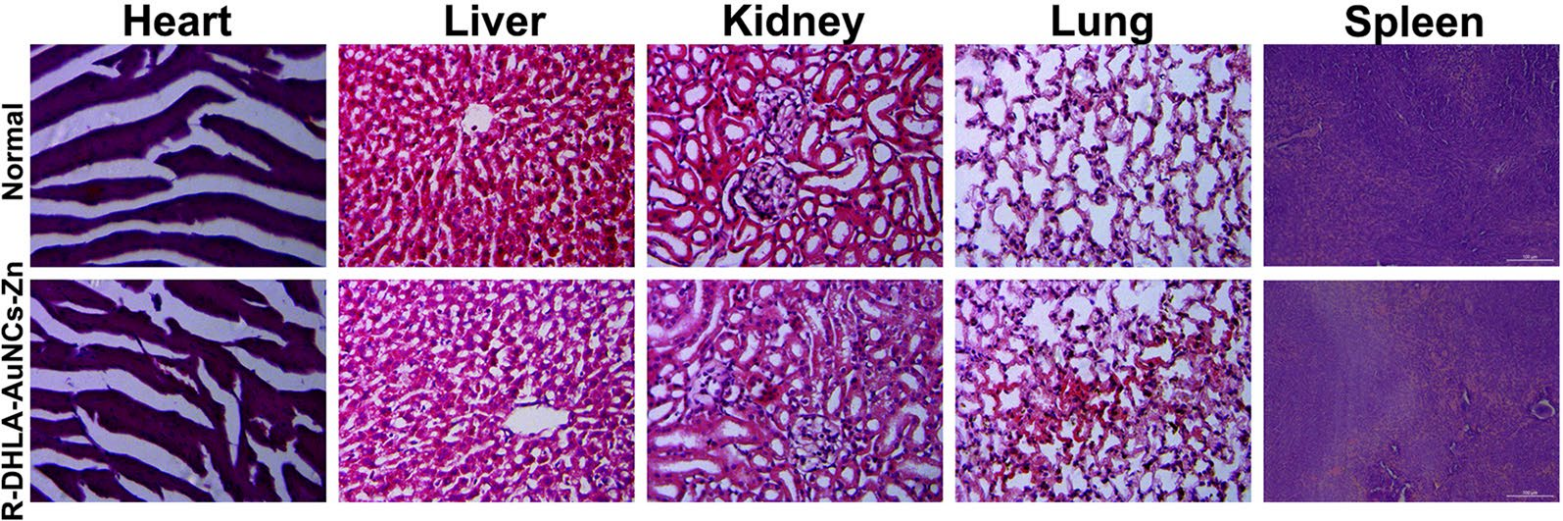
In vivo toxicity evaluation of R-DHLA-AuNCs-Zn on the 28th day in SCI rats compared to normal control rats. Scale bar = 100 μm. (n = 6/group)

After 28 days of treatment, the toxicity of R-DHLA-AuNCs-Zn to the organs was investigated by histopathological analysis (Fig. 9). No obvious change was observed from the H&E stained major organs of the heart, liver, spleen, lung, and kidney. And no apparent histopathological abnormalities or lesions were observed in each organ.

In the clinical diagnosis of infectious or traumatic diseases, the biomarkers such as C-reactive protein (CRP) reflect the severity of the inflammation and disorder in vivo. To investigate the safety and therapy effects of R-DHLA-AuNCs-Zn to SCI rat models, the peripheral blood cells and biochemical analysis through automatic hematology testing systems were investigated. The SCI rats had serious inflammation, which was indicated by the increase of CRP (Additional file 1: Fig. S8a), Alkaline phosphatase (ALP, Additional file 1: Fig. S8b), Alanine transferase (ALT, Additional file 1: Fig. S8c), WBCs (Additional file 1: Fig. S8d), the ratio of monocyte (Additional file 1: Fig. S8e) and platelets (PLTs, Additional file 1: Fig. S8f) compared to normal rats. Aspartate aminotransferase (AST) showed indifferent change (Additional file 1: Fig. S8g). Moreover, a remarkable decrease in RBCs (Additional file 1: Fig. S8h) and Hemoglobin (Hb, Additional file 1: Fig. S8i) was found. On the other hand, the administration of R-DHLA-AuNCs-Zn successfully recovered these factors to healthy conditions. These results indicated that R-DHLA-AuNCs-Zn showed insignificant in vivo toxicity, which simultaneously influenced blood-derived immune cells, showing antiinflammation effects on the SCI model.

## Discussion

SCI is a medical emergency that is motive for the ischemia and hypoxia of the injured spinal cord [1]. M/Ms were reported participating in inflammatory cascade after SCI, including neuroinflammation, ROS production, and secondary injuries [1]. However, the correlation between M/Ms and the ASIA motor score at final follow-up in SCI patients remains unclear. Herein, we found the SCI symptoms were relieved and the ASIA motor score was promoted, proving their close relationship among these therapeutic effects. The immunosuppressive strategy promoted significant limb function recovery, which was achieved by intravenous injection of R-DHLA-AuNCs-Zn. In addition, the administration of R-DHLA-AuNCs-Zn could produce antioxidant and anti-inflammatory effects in vivo and in vitro. R-DHLA-AuNCs-Zn transformed M1 as M2, especially M/Ms in lesions related to functional recovery after SCI. This also achieved other therapeutic effects, including stronger apoptosis inhibition and neuron protective activity than various other nanomedicines [27–30]. In addition, R-DHLA-AuNCs played a role in neutralizing extracellular inhibitors at the lesion site bridging the lesion site with permissive grafts. These comprehensive treatment effects may ultimately benefit patients with SCI. Therefore, R-DHLA-AuNCs-Zn are promising to provide practical value for clinical applications.

## Conclusion

In summary, R-DHLA-AuNCs-Zn were successfully fabricated for recovering SCI symptoms for the rat models by the comprehensive therapeutic effects. Especially, we found that R-DHLA-AuNCs induced M2 polarization, which might benefit function recovery by reducing neuronal apoptosis and lesion size. R-DHLA-AuNCs-Zn also promoted neuron survival, decreased inflammations, and suppressed oxidation stresses. The R-DHLA-AuNCs-Zn had no long-term toxicity and showed remarkable immune-suppressing responses. We proposed that the better treatment effects of zinc-modified R-DHLA stabilized AuNCs for SCI were attributed to the R-chiral surface, though further investigations will perform to understand the deep mechanisms. This work opens the avenue for the easy and safe treatment of human SCI using NCs-based materials, which will be used in future research.

## Supplementary Information

**Additional file 1:** Including demographic and clinical characteristics of SCI Subjects, TEM-EDS of AuNCs, quantification of mRNA expression and ROS assays in vivo, and R-DHLA-AuNCs-Zn, primer sequences used for quantitative real-time PCR.

## Data Availability

Most of the datasets supporting the conclusions of this article are included within this article. The datasets used or analyzed during the current study are available on reasonable request.

## Abbreviations

SCI: Spinal cord injury
CNS: Central nervous system
M/Ms: Microglia/macrophages
ROC: Receiver-operating characteristics
AuNCs: Gold nanoclusters
DHLA: Dihydrolipoic acid
ROS: Reactive oxygen species
TEM: Transmission electron microscopy
EDS: Energy-dispersive X-ray spectroscopy
ICP-MS: Inductively coupled plasma mass spectrometry
MTT: (4,5)-Dimethylthiahiazo (-z-y1)-3,5-di-phenytetrazoliumromide
BBB: Basso, Beattie, and Bresnahan
ASIA: American Spinal Injury Association
DMEM: Dulbecco’s modified Eagle medium
FBS: Fetal bovine serum
PS: Penicillin–streptomycin
H&E: Hematoxylin and eosin
RPS18: Ribosomal protein S18
BSA: Bovine serum albumin
RBCs: Red blood cells
WBCs: White blood cells
AUC: Area under the curve
IL-1β: Interleukin 1 beta
TNF-α: Tumor necrosis factor-alpha
LPS: Lipopolysaccharide
CAT: Catalase
GPx: Glutathione peroxidase
HNE: Hydroxynonenal
MDA: Malondialdehyde
ALP: Alkaline phosphatase
ALT: Alanine transferase
PLTs: Platelets
AST: Aspartate aminotransferase
Hb: Hemoglobin
