# Supplementary material for: Therapy of spinal cord injury by zinc modified gold nanoclusters via immune-suppressing strategies
Source: J Nanobiotechnology. 2021 Sep 20;19:281. doi: 10.1186/s12951-021-01035-8 (PMC8454132; doi:10.1186/s12951-021-01035-8)
Supplement: Supplementary file 1 — Additional file 1: Including demographic and clinical characteristics of SCI Subjects, TEM-EDS of AuNCs, quantification of mRNA expression and ROS assays in vivo, and R-DHLA-AuNCs-Zn, primer sequences used for quantitative real-time PCR. [file 12951_2021_1035_MOESM1_ESM.doc]

**Therapy of spinal cord injury by zinc modified gold nanoclusters via** **immune-suppressing strategies**

Sen Lin^1,#^, Dan Li^2,#^, Zipeng Zhou^1^, Chang Xu^1^, Xifan Mei^1,*^, He Tian^2,*^

^1^Department of Orthopaedic Surgery, First Affiliated Hospital, Jinzhou Medical University Jinzhou 121000, P. R. China

^2^Department of Basic Science, Jinzhou Medical University, Jinzhou 121000, P. R. China

^#^S. Lin and D. Li contributed equally to this work.

*E-mail: meixifan@jzmu.edu.cn

**Table S1.** Primer sequences used for quantitative real-time PCR.

| Gene | Forward Primer (5’ to 3’) | Reverse Primer (5’ to 3’) |
| --- | --- | --- |
| IL-1β | CCTGTGCTGTCGGACCCATA | CAGGCTTGTGCTCTGCTTGTGA |
| TNF-α | GTGCTTGCTGGCTCACAGTTA | GGTTGGTGTACCCCCATTCA |
| Arg-1 | CTCCAAGCCAAAGTCCTTAGAG | AGGAGCTGTCATTAGGGACATC |
| IL-1ra | CATGACTGCCCATTGTTGAG | AGGGCAGAAGCCTAGGAAG |
| RPS18 | GCAATTATTCCCCATGAAG | GGCCTCACTAAACCATCCAA |

**Table S2.** Demographic and Clinical Characteristics of Subjects

| Subjects | N/(n) | Sex | | Age(Y) | ASIA | | | |
| --- | --- | --- | --- | --- | --- | --- | --- | --- |
|  |  | Men | Women |  | A | B | C | D |
| SCI patients | 114 | 83 | 32 | 55.2±12.7 | 29 | 25 | 46 | 14 |
| Paraplegia | 63 | 47 | 16 | 55.3±13.4 | 16 | 17 | 25 | 5 |
| Tetraplegia | 51 | 35 | 16 | 55.1±11.9 | 13 | 8 | 21 | 9 |
| Control | 90 | 48 | 42 | 45.1±10.2 | NA | NA | NA | NA |

**Table S3.** TEM-EDS of DL-DHLA-AuNCs-Zn and R-DHLA-AuNCs-Zn

|  | DL-DHLA-AuNCs | | R-DHLA-AuNC-Zn | |
| --- | --- | --- | --- | --- |
| Element | wt% | Atom ratio% | wt% | Atom ratio% |
| C | 72.25 | 87.34 | 84.90 | 92.84 |
| O | 9.31 | 8.45 | 7.20 | 5.91 |
| S | 7.34 | 3.32 | 1.84 | 0.75 |
| Zn | 0.46 | **0.10** | 0.67 | **0.13** |
| Au | 10.65 | 0.78 | 5.40 | 0.36 |
| Zn% |  | 11.3 | 26.5 |  |
| Total: | 100.00 | 100.00 | 100.00 | 100.00 |


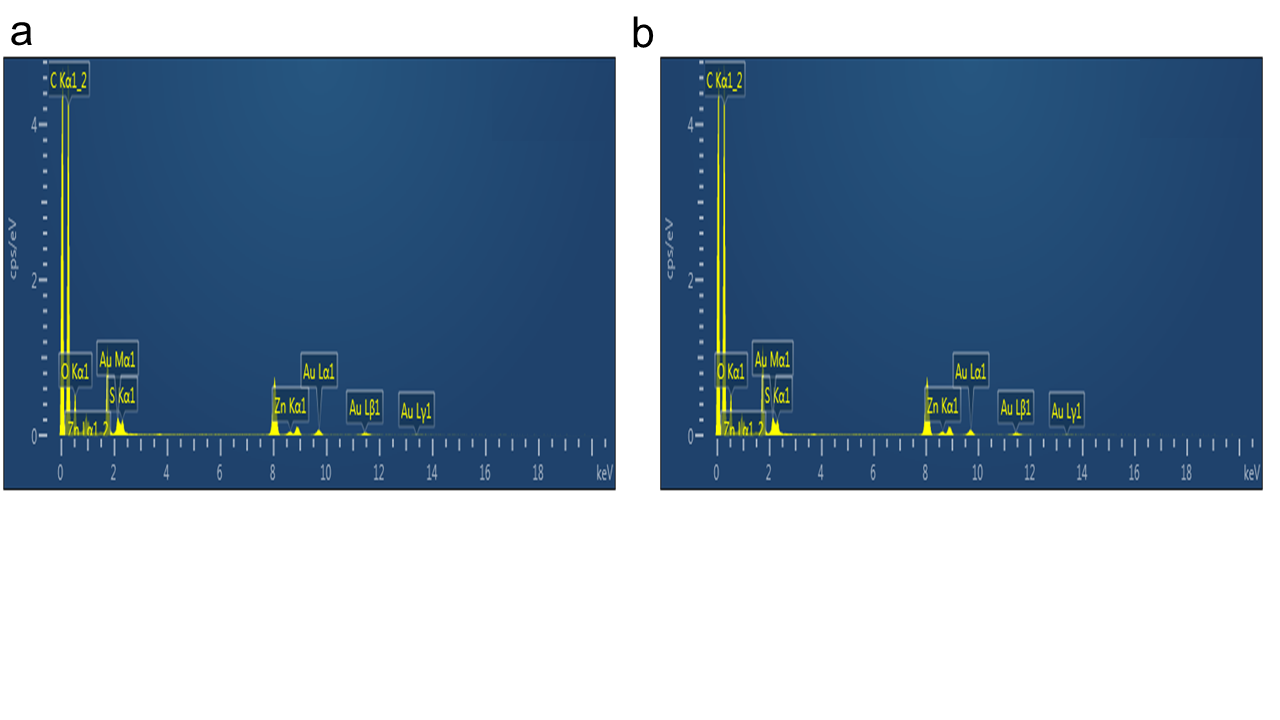


**Figure S1.** TEM-EDS of DL-DHLA-AuNCs-Zn and R-DHLA-AuNCs-Zn


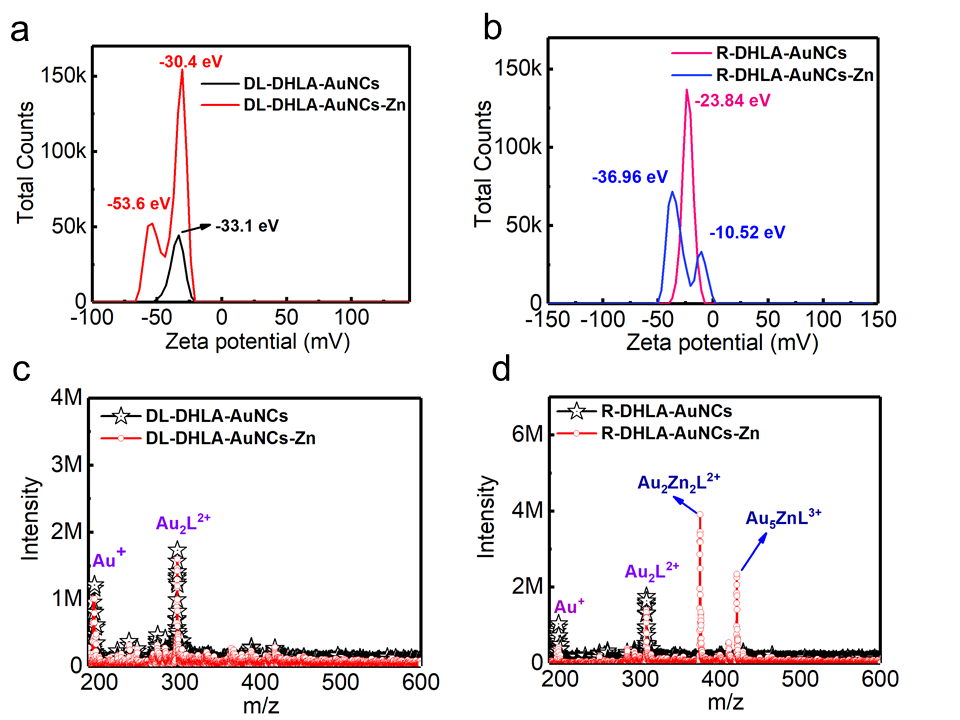


**Figure S2.** Zeta potential (a, b) and ESI-MS (c, d) of DL-DHLA-AuNCs (a, c), DL-DHLA-AuNCs-Zn (a, c), R-DHLA-AuNCs (b, d) and R-DHLA-AuNCs-Zn (b, d).


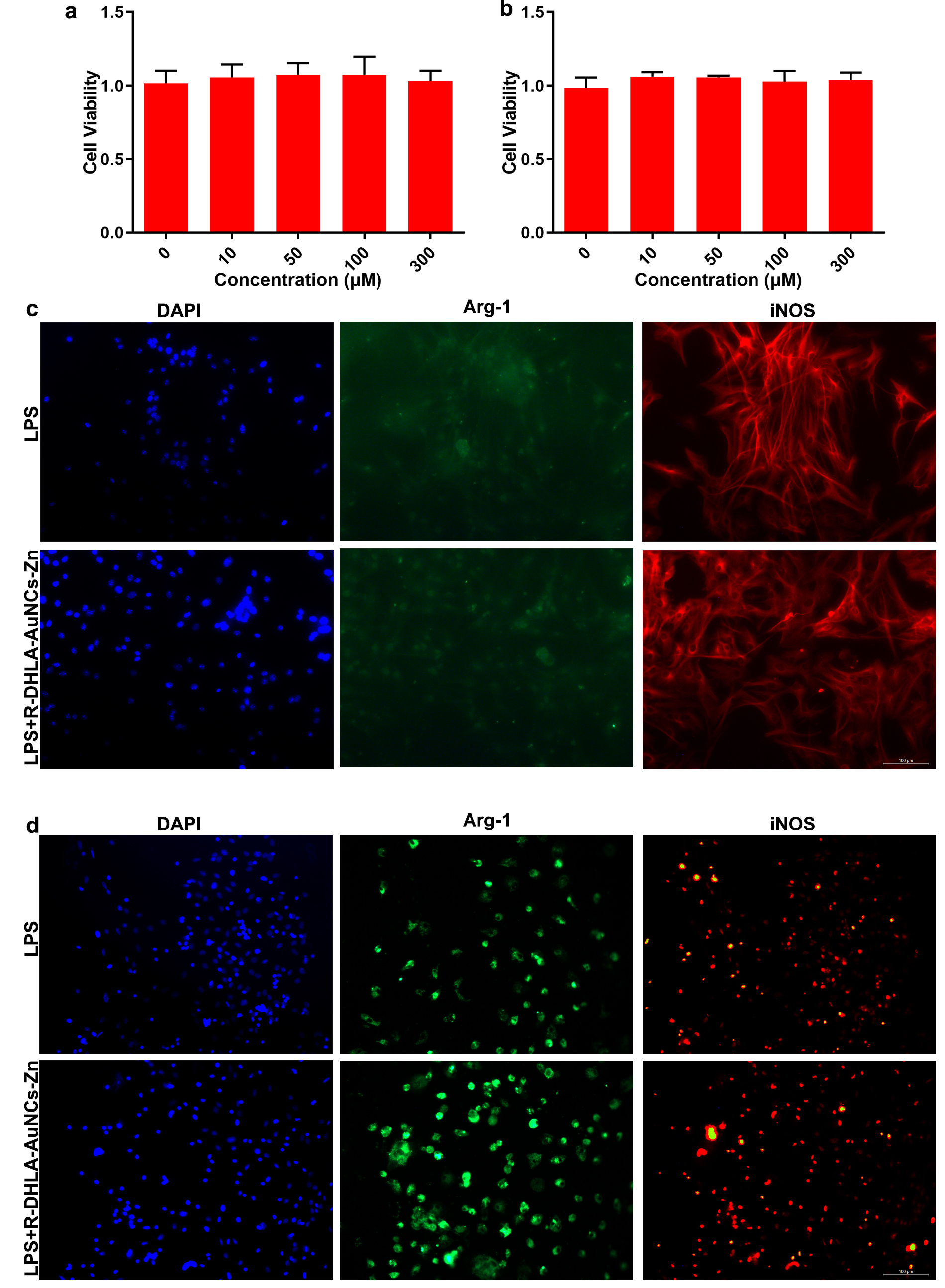


**Figure S3.** R-DHLA-AuNCs-Zn had indifference to other glia cells. The cell viabilities of R-DHLA-AuNCs-Zn in MA-c astrocyte cells (a) and MO3.13 oligodendrocyte cells (b) The immunofluorescence staining (e) of iNOS and Arg-1 in MA-c astrocyte cells (c) and MO3.13 oligodendrocyte cells (d) treated with LPS or LPS+R-DHLA-AuNCs-Zn. Scale bar=100 μm. Data presented as mean ± SD. (n = 6 /group).

**
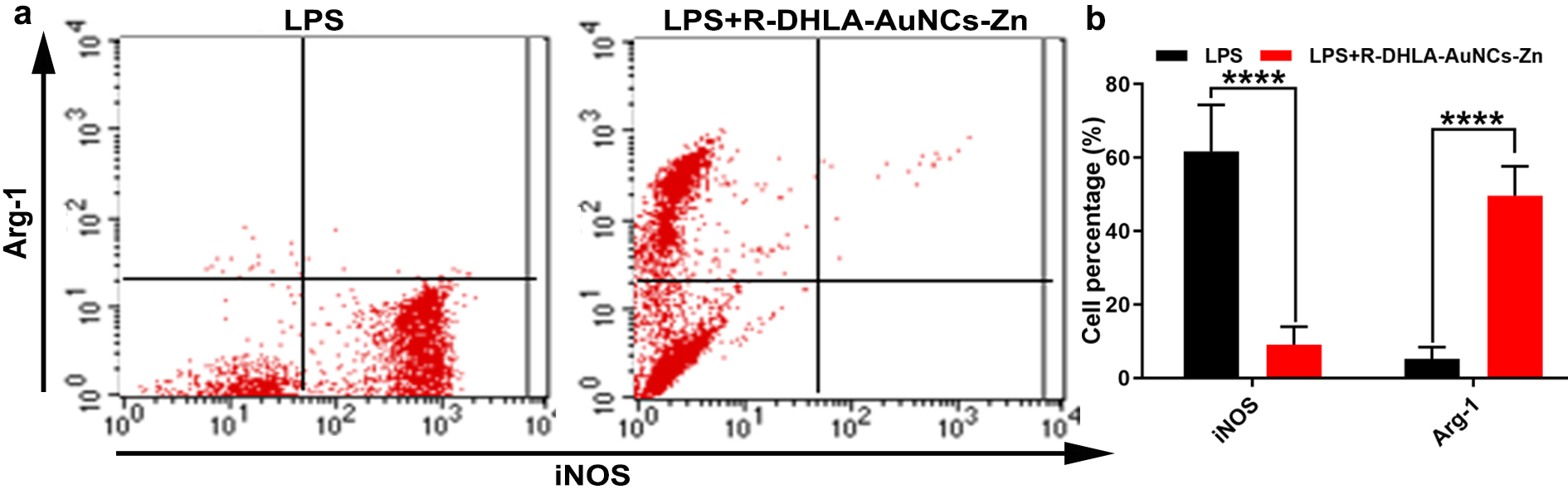
**

**Figure S4.** The flow analysis (a) and the corresponding quantification of iNOS and Arg-1 (b).


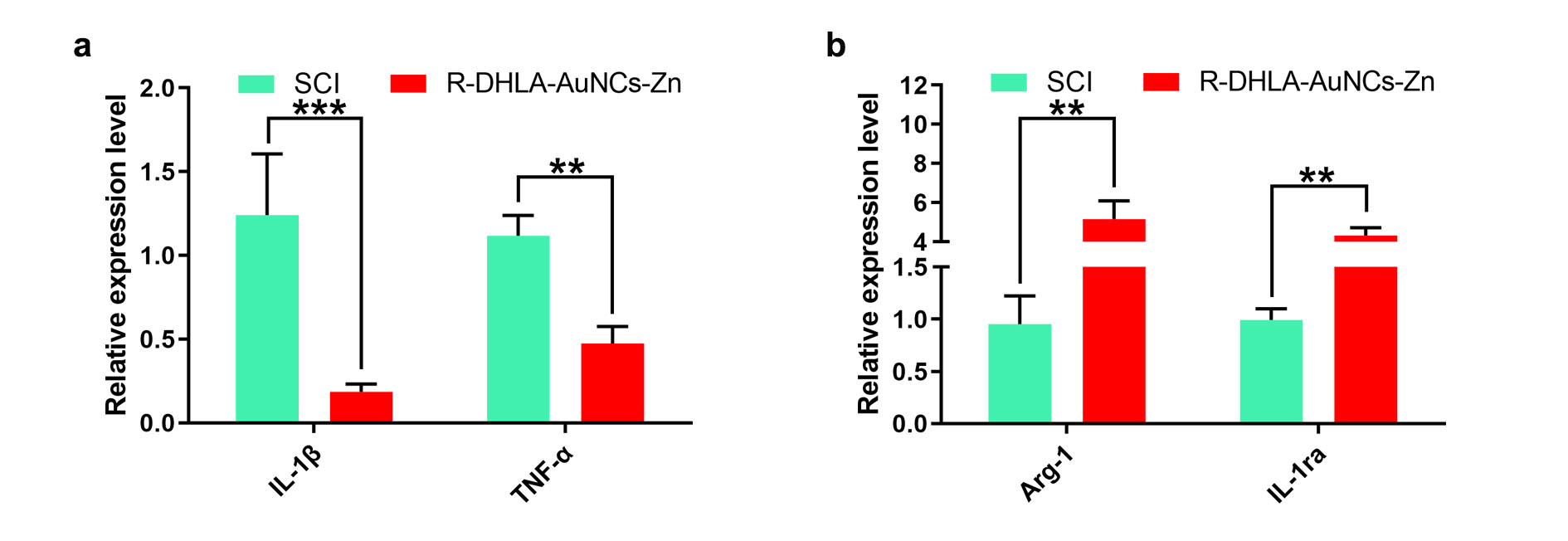


**Figure S5.** R-DHLA-AuNCs-Zn promoted M2 polarization after SCI. The expression of IL-1β, TNF-α (a), Arg-1 and IL-1ra (b) in RT-PCR analysis at 7 days after SCI.**P < 0.01;***P < 0.001.Data presented as mean ± SD. (n = 6 /group).

**
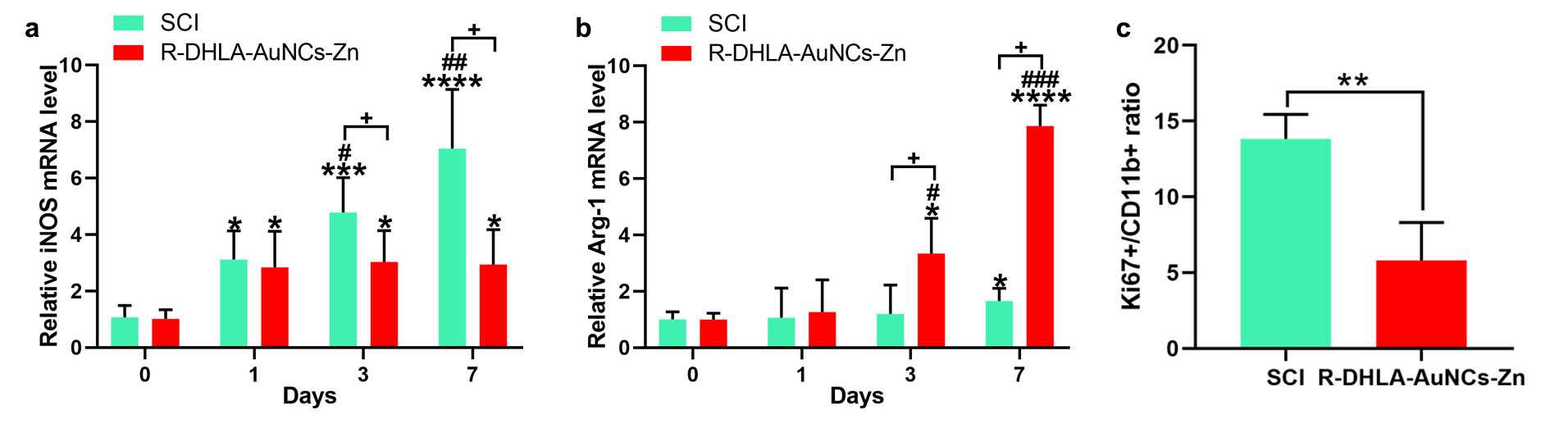
**

**Figure S6.** R-DHLA-AuNCs-Zn induced M2 markers after SCI. Quantification of expression of M1: iNOS (a) or M2 markers: Arg-1 (b) in the injured spinal cord of SCI group or R-DHLA-AuNCs-Zn group at 1, 3, and 7 days during the acute course of SCI. Quantification of expression of Ki67 in the injured spinal cord of SCI group or R-DHLA-AuNCs-Zn group at 7 days after SCI. Data are mean ± SD (n=6); *, significant difference compared to day 0 (*, p<0.05; **, p< 0.01; ***, p<0.001; ****, p<0.0001); #, compared to day 1 (#, p<0.05; ##, p< 0.01; ###,p< 0.001); +, significant difference (p< 0.05) between SCI and R-DHLA-AuNCs-Zn.

**
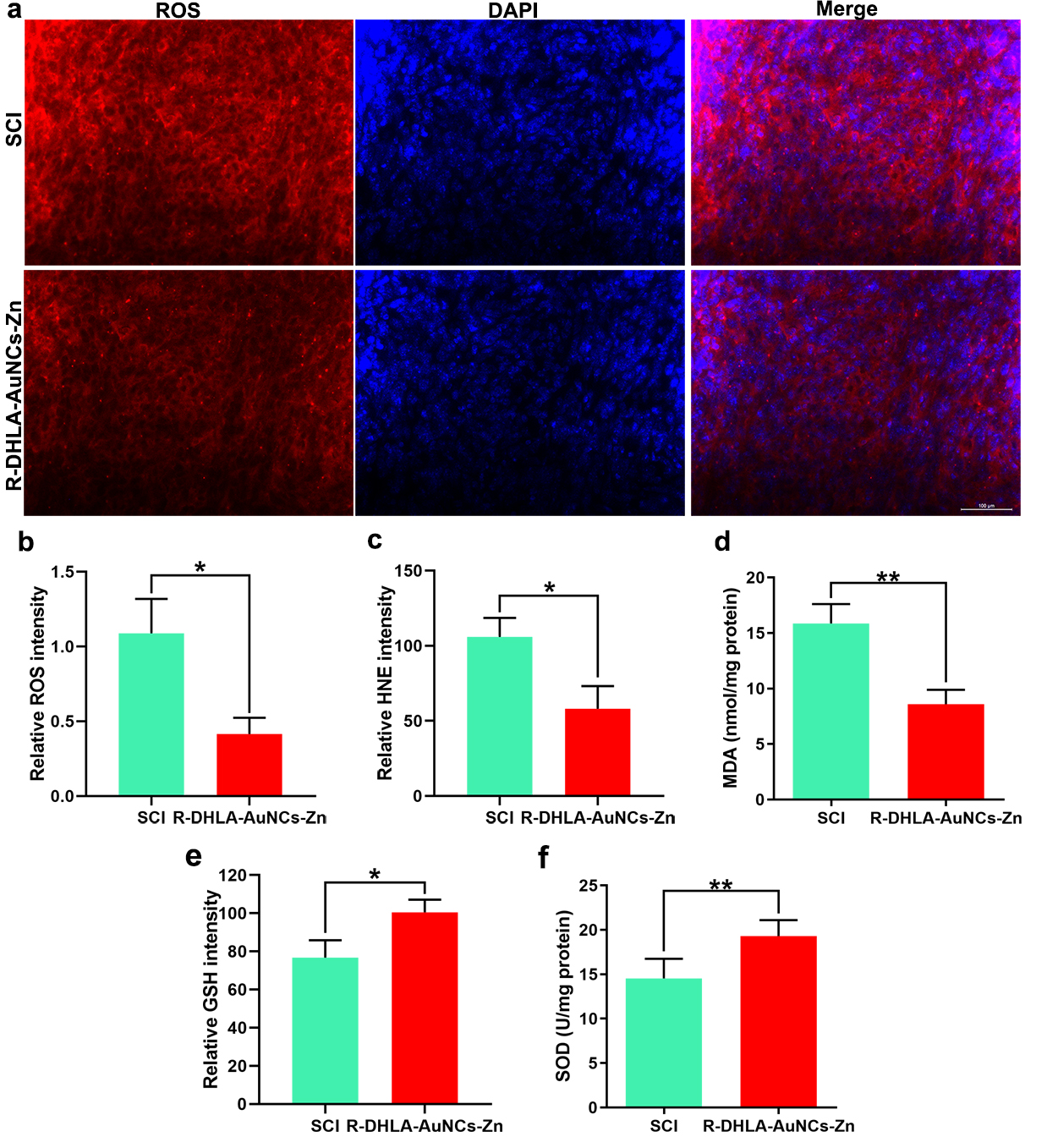
**

**Figure S7.** R-DHLA-AuNCs-Zn inhibited SCI-induced peroxidation. ROS staining (a) and representative quantification (b) of the spinal cord of SCI group or R-DHLA-AuNCs-Zn group at 7 days after SCI. Representative quantification of the oxidant enzymes: HNE (c) and MDA (d), and antioxidant proteins: GSH (e) and SOD (f) activity in the spinal cord of SCI group or R-DHLA-AuNCs-Zn group at 7 days after SCI. Data are mean ± SD (n=6); *, significant difference compared to SCI (*, p<0.05).

**
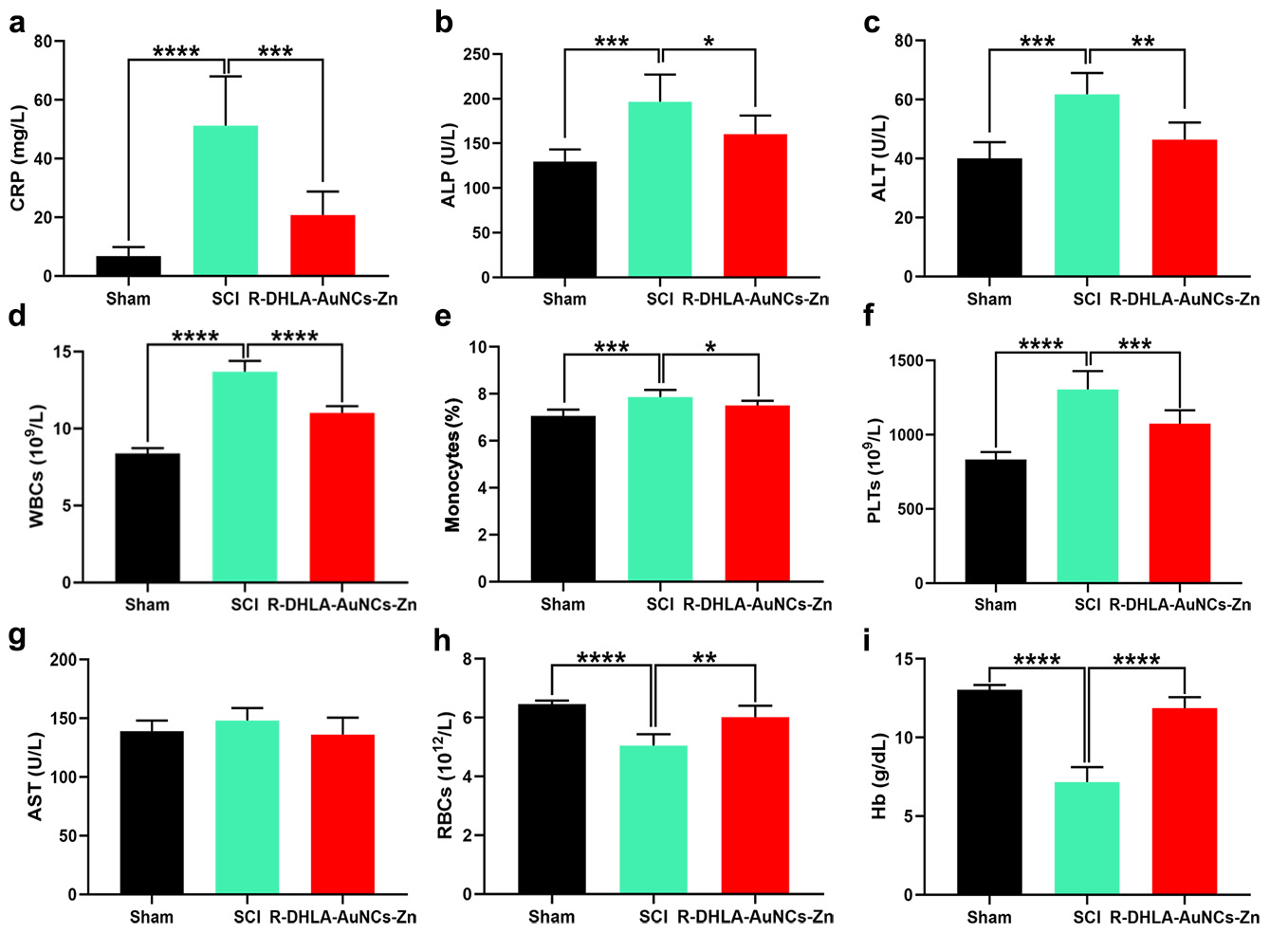
**

**Figure S8.** Administration of R-DHLA-AuNCs-Zn had biological safety in SCI mice. Representative quantification of the CRP (a), ALP (b), ALT (c), White blood cells (d), monocytes (e), Platelets (f), AST (g), Red blood cells (h), Hb (i) content in the spinal cord of SCI group or R-DHLA-AuNCs-Zn group at 28 days after SCI. Data are mean ± SD (n=6); (*, p<0.05; **, p< 0.01; ***, p<0.001; ****, p<0.0001).
